# MICROSTATELAB: The EEGLAB Toolbox for Resting-State Microstate Analysis

**DOI:** 10.1007/s10548-023-01003-5

**Published:** 2023-09-11

**Authors:** Sahana Nagabhushan Kalburgi, Tobias Kleinert, Delara Aryan, Kyle Nash, Bastian Schiller, Thomas Koenig

**Affiliations:** 1 Children’s Hospital Los Angeles, The Saban Research Institute, Los Angeles, CA 90027 USA; 2 Laboratory for Biological Psychology, Clinical Psychology, and Psychotherapy, Albert-Ludwigs-University of Freiburg, Stefan-Meier-Straße 8, 79104 Freiburg, Germany; 3 Department of Ergonomics, Leibniz Research Centre for Working Environment and Human Factors, Ardeystr. 67, 44139 Dortmund, Germany; 4 Department of Psychology, University of Alberta, Edmonton, AB T6G 2E9 Canada; 5 Translational Research Center, University Hospital of Psychiatry and Psychotherapy, University of Bern, CH-3000 Bern, Switzerland; 6 Department of Neurobiology, Care Sciences and Society, Center for Alzheimer Research, Division of Clinical Geriatrics, Karolinska Institutet, Huddinge, Sweden

**Keywords:** EEG microstates, EEGLAB, Toolbox, Tutorial, Resting state

## Abstract

Microstate analysis is a multivariate method that enables investigations of the temporal dynamics of large-scale neural networks in EEG recordings of human brain activity. To meet the enormously increasing interest in this approach, we provide a thoroughly updated version of the first open source EEGLAB toolbox for the standardized identification, visualization, and quantification of microstates in resting-state EEG data. The toolbox allows scientists to (i) identify individual, mean, and grand mean microstate maps using topographical clustering approaches, (ii) check data quality and detect outlier maps, (iii) visualize, sort, and label individual, mean, and grand mean microstate maps according to published maps, (iv) compare topographical similarities of group and grand mean microstate maps and quantify shared variances, (v) obtain the temporal dynamics of the microstate classes in individual EEGs, (vi) export quantifications of these temporal dynamics of the microstates for statistical tests, and finally, (vii) test for topographical differences between groups and conditions using topographic analysis of variance (TANOVA). Here, we introduce the toolbox in a step-by-step tutorial, using a sample dataset of 34 resting-state EEG recordings that are publicly available to follow along with this tutorial. The goals of this manuscript are (a) to provide a standardized, freely available toolbox for resting-state microstate analysis to the scientific community, (b) to allow researchers to use best practices for microstate analysis by following a step-by-step tutorial, and (c) to improve the methodological standards of microstate research by providing previously unavailable functions and recommendations on critical decisions required in microstate analyses.

## Introduction

Brain activity is innately spontaneous and self-organizing. Most neural activity within the brain is internal and independent of sensory input or motor output. Similarly, we are mostly able to maintain a coherent stream of mental representations, not only because but even against a constant influx of sensory stimulation. Given that such self-organizing brain activity also constitutes the material basis of mental states, it follows that one can use the study of spontaneous brain activity to study self-organizing mental states in health and disease, gain a biological understanding of the variations of physiological states, and eventually obtain the ability to intervene with behavioral or pharmacological treatment where appropriate. The analysis of spontaneous EEG using the microstate framework is one such approach. EEG microstate analysis assumes that spontaneous brain activity is largely organized in sub-second time periods of large-scale in- and anti-phase states of oscillations (Michel and Koenig 2018) of cortical excitability that define the brain’s overall mode of information integration. In addition, microstate analysis typically assumes that there is only a small but rather universal and prototypical set of such states and that these states can be identified as periods of quasi-stable scalp field topography in the EEG. Microstate analysis draws basic support for these assumptions from the fact that in spontaneous EEG data, there are easily observable brief periods (40–120 ms) of stable field configuration with periodic polarity reversals (Lehmann 1990; Lehmann et al. 1987). From a physics perspective, in- and anti-phase oscillations of large-scale brain sources are the most plausible explanation for the phenomenon of synchronous polarity reversal of spontaneous EEG fields (Michel and Koenig 2018). Perhaps most relevant, there is a rapidly increasing body of empirical studies that shows systematic associations of variations in EEG microstates with variations in mental states [for reviews see (Khanna et al. 2015; Michel and Koenig 2018)].

Microstate analysis is about identifying and quantifying a limited set of global functional brain states defined by a common (albeit periodically reversing) scalp field of the ongoing EEG that is attributed to one of these brain states through volume conduction of the active sources to the scalp. Microstate analysis, therefore, typically involves the identification of predominant classes of EEG scalp fields (microstate maps) that likely represent the predominant set of global functional brain states present during the individual recordings. Once these individual microstate maps have been identified, they must be sorted in a similar manner across subjects to extract a set of mean microstate maps that represents a common set of brain functional states observable in the experimental group and condition. This set of mean microstate maps can then be used to infer functional significance by querying empirical findings associated with spatially similar maps in other studies. In addition, the mean template maps can be competitively fitted to the individual EEGs, which yields a sequence of assignments of EEG time to these microstate classes. Individual microstates can then be defined as continuous time periods assigned to the same class and used to extract individual quantifiers of EEG microstates in the given EEG data, such as their mean duration, frequency of occurrence, and percent time covered as a function of microstate class. The extracted features can then be statistically tested for differences between groups and/or conditions to address possible alterations in the recruitment of particular global brain functional states.

Several toolboxes are available for microstate analysis (Brunet et al. 2011; Férat et al. 2022; Poulsen et al. 2018; Tait and Zhang 2022; von Wegner and Laufs 2018). There is substantial variability in the analytical approaches applied to microstates. This reflects a limited standardization and challenges in comparing datasets across studies. To address the analytical challenges, here, we introduce a thoroughly updated version of the first *EEGLAB toolbox for resting-state microstate analysis,* or MICROSTATELAB. This toolbox provides to the neuroscience community a curated, standardized and validated open access, software pipeline for the identification, visualization, and quantification of EEG microstates. The development of the toolbox has several goals: (1) to enable researchers from different fields, even with no previous experience with EEGLAB or MATLAB, to conduct microstate analyses by following a step-by-step tutorial; (2) to improve the current methodological standards of microstate research by providing and discussing recommendations for critical decisions that are necessary to conduct accurate microstate analysis across studies.

This manuscript is structured in four parts. First, we provide an overview of the microstate analysis. Second, we provide recommendations on critical decisions across the various steps of the analysis. Third, we provide a step-by-step tutorial using the graphical user interface (GUI) as well as batch processing using a sample script on how to use the toolbox using the sample dataset. Lastly, we discuss the advantages and disadvantages of the microstate approach and future goals of microstate research.

## Overview of Microstate Analysis

**Fig. 1:**
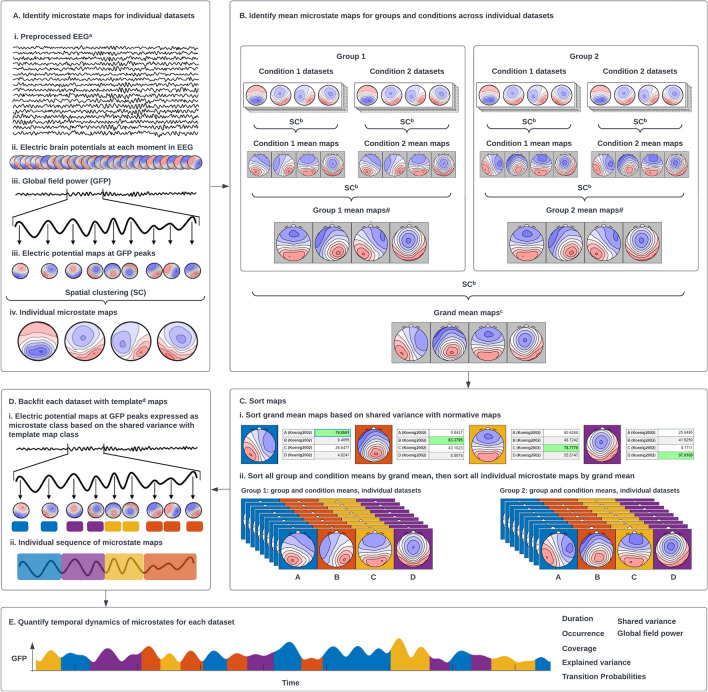
Overview of EEG microstate analysis. **A** Identification of microstate map at the subject level. ^a^Refer to Nagabhushan Kalburgi et al., this issue. **B** Identification of mean and grand mean maps. ^b^Modified spatial sorting. **C** Hierarchical sorting of grand mean, mean, and subject level maps. ^c^Refer to Koenig et al., Metamaps paper this issue for more information on comparing your maps with other published maps. **D** Backfitting template maps onto GFP peaks of EEG at subject level. ^d^Refer to (Murphy et al. 2022) which demonstrated that extracting temporal features using group mean maps as templates led to faulty results. **E** Extraction of temporal parameters at subject level

The following section provides a brief outline and rationale of the typical steps of a microstate analysis in their standard sequence (Fig. 1). Important choices necessary for these steps are then discussed in the subsequent section.

### Identify Individual Microstate Maps

As outlined above, in the first step of microstate analysis, individual microstate maps need to be identified that represent the activation of specific brain networks of coherent activation, which occurs across brief time periods (Fig. 1A). This is achieved by applying a cluster analysis on the time series of the electric potential field map topographies of individual preprocessed EEG recordings. These individual maps are clustered based on their topographic similarity, which are then assigned to the same class. Note that this clustering permits polarity reversals, which is implemented by using the squared spatial correlation coefficient as similarity measure and by using the first principal component instead of the average to obtain cluster template maps (Pascual-Marqui et al. 1995). The toolbox offers a convenient pipeline to identify various numbers of individual microstate maps across multiple subjects in a single step. Studies have described cluster number solutions ranging between 4 and 7 classes.

### Averaging and Sorting of Microstate Maps

As the initial clustering of the individual EEG datasets yields individual microstate maps in no particular order, the next step of the analysis is to reorder the individual microstate maps in a way that maximizes their shared variance across subjects. Depending on the question at hand, this can be done for all the subjects’ EEGs jointly or separately for different groups and/or conditions. This sorting is obtained through a second-level clustering of the individual microstate maps into grand-mean maps under the constraint that for each individual set of microstate maps, there is a one-to-one relationship to the grand-mean microstate maps, i.e., all grand-mean microstate maps need to be assigned to exactly one individual microstate map and vice versa. Thus, based on this second-level clustering of the individual template maps, meaningful group, condition, or sample mean microstate maps are obtained (Fig. 1B), and the individual microstate maps can be commonly ordered in accordance with their assignment to the grand-mean microstate maps. Note that if several mean microstate maps (e.g., group mean, condition mean, grand mean of group and condition means) are obtained, the sorting needs to be repeated to allow for the computation of a grand mean microstate map template (Fig. 1B). The sorting of the individual microstate templates needs to be updated accordingly.

Finally, microstate classes in the literature are often labeled in a specific way based on their topography (Custo et al. 2017; Koenig et al. 2002) which relates to the functional associations of these microstates. Therefore, to compare new findings against published literature, it is useful to order and label the microstate template maps in a way that refers to these published templates (Fig. 1C). The grand mean template maps can be sorted based on the similarity to published maps, followed by the sorting of individual and group-level template maps by the grand mean maps (Fig. 1C). If the topographical characteristics of the grand mean template maps are sufficiently similar to the published templates and share a high spatial correlation, their functional roles can be considered to be similar (see also Koenig et al., this issue (Metamap paper)). Therefore, the toolbox offers the possibility to quantify and visualize similarities in the form of spatial correlations on a multi-dimensional scale. If the grand mean template maps do not sufficiently resemble the published template maps, the data may represent features not resolved in previous studies or may require further preprocessing. If a cluster number was chosen that has not been used in previous research, it is still possible to sort the maps in a meaningful order by comparing the topographies with template maps using a different number of clusters.

The toolbox offers a full complement of tools to obtain mean microstate maps across subjects after optimizing their sequence for maximal shared variance, to combine group mean template maps into grand mean template maps, to visualize the obtained results and compare them to published templates and update the order of individual template maps based on a representative mean template.

### Outlier Detection

There is considerable interindividual variability in the appearance of microstate networks, and some individual microstate maps may lack any correspondence to the prototypical network classes, including the example provided here. There are certain artifacts (eye movements, blinks, high impedance in electrodes) that produce EEG signals that may falsely be identified as brain microstates. Thus, if an individual’s microstate maps clearly diverge from their usual appearance in the literature or in one’s sample, this may be due to low EEG-quality. Inspecting microstate maps is, therefore, a critical step to identify and eliminate artifacts that elude the preprocessing procedures and thereby improve data quality. The toolbox offers two options—data quality check and outlier detection. The data quality check option detects datasets with aberrant channels that produce patchy maps, which are resolved by channel interpolation. The outlier detection option detects topographies with large differences from the other members of the group or false states. As the outlier detection must be protected from falsely identifying differently ordered individual microstates templates, the outlier detection can only be applied after correctly sorting the templates. If a dataset contains an outlier in any class, it should be further inspected and re-preprocessed. If the datasets continues to exhibit topographic outliers in any class after further cleaning, the entire dataset should be excluded. To account for such exclusions, the mean and grand mean maps must be regenerated.

### Backfitting and Quantification of Microstate Dynamics

The next step of the microstate analysis is to quantify, visualize and export the temporal dynamics of individual recordings that comprise the dataset, which can be used for further statistical analyses with standard statistical software. For this step, all initial individual electric potential field maps are assigned to the best-fitting microstate template map (backfitting), resulting in a continuous sequence of microstate maps in each individual (Fig. 1D). Then, features of these identified microstates are extracted. Typical microstate dynamics features include the average duration in milliseconds, the average number of occurrences per second, the average coverage of the EEG signal in percent for each microstate class, the global field power of microstate classes, transition probabilities, spatial correlation with published templates, explained variance of each microstate class, and the global explained variance of all microstate classes combined (Fig. 1E). An overview of the microstate features extracted by the toolbox and their current understanding is listed in Table 1. The toolbox extracts all of these features across the entire sample in a single step, plots them, and exports them in a variety of formats that can be directly used by common spreadsheet and statistics packages.

**Table 1:** Overview of the temporal dynamics of EEG microstates quantified after backfitting and their interpretability in terms of brain network activity

Microstate parameters utilized for statistical analysis	How is it measured	Potential interpretation regarding underlying neural network processing
Duration of class X	Average duration of all microstates belonging to the microstate class X	Stability of neural networks represented by microstate class X
Occurrence of class X	Frequency (per second) that microstates of class X were observed	Frequency of activating neural networks represented by class X
Coverage of class X	Percentage of total time a microstate class is present	Percentage of time that the brain spent in neural networks represented by class X
MeanGFP of class X	Mean Global Field Power of all time periods assigned to microstate class X	Mean strength of all sources that were active while neural networks represented by microstate class X were predominant
Transition probabilities X $\to$ Y (OrgTM X $\to$ Y)	Among all transitions between microstates, percentage of the number of times that there is a transition from microstate class X to microstate class Y. Note that it is by definition impossible for a microstate to transition to itself, thus all transition probabilities of type X $\to$ X are by definition inexistent. Note also that these transition probabilities will depend on the relative occurrences of particular microstate classes	Tendency of neural networks represented by class X to activate neural networks represented by class Y
Adjusted transition probabilities (DeltaTM X $\to$ Y)	Differences in transition probabilities against the transition probabilities as they are expected under randomness, given the relative occurrences of particular microstate classes This is calculated as follows: $\mathrm{DeltaTM}(\mathrm{X}\to \mathrm{Y})=\left(\frac{OrgTM(X\to Y)-ExpTM(\mathrm{X}\to \mathrm{Y})}{ExpTM(\mathrm{X}\to \mathrm{Y})}\right) \times 100$, where ExpTM(X $\to \mathrm{Y}$) is computed according to Lehmann et al. 2005	Relative preference or resistance to enter microstate Y from microstate X, against chance level. Addresses the question if there are particular rules (or a “syntax”) in the chain of microstates
Explained variance of class X	Percentage of the total variance explained by a given microstate class	Percentage of overall neural activity attributed to neural networks represented by class X
Mean duration	Average period of microstate activation irrespective of class	Overall Stability of neural networks represented by all microstate classes
Mean occurrence	Average rate of microstate activation irrespective of class	Inverse of mean duration
Total time	The sum of all microstate durations across epochs	None, this is merely to know how much data was analyzed
Total explained variance	The overall proportion of the variance of the EEG that is explained by all microstate classes	Goodness of fit of the chosen microstate template maps with the analyzed EEG

Backfitting and quantification can be performed with individual template maps, grand mean or published template maps. Backfitting and quantifying according to individual template maps has the advantage of providing an optimal fit between the chosen template and their respective EEG data. However, using a different template for each subject decreases the comparability of the exported individual microstate characteristics (Kleinert under review), because the spatial variance in the individual template maps is likely to increase the variance in the extracted features. Therefore, many studies have backfitted the data using grand mean template maps, which guarantees a consistent assignment across subjects and, thus, optimal comparability of the microstate characteristics extracted. Note that fitting on group-level maps is not recommended as it can introduce false positive findings (Murphy et al. 2022). For a conservative statistical analysis, feature extraction should thus always be based on a single common mean template, or on individual template maps that were sorted according to such a common template.

### Compare Microstate Maps

Groups and conditions may differ not only in the relative presence, occurrence, or duration of particular microstate classes but also in the spatial distribution of their typical microstate maps. The toolbox currently offers an interface to Ragu (Habermann et al. 2018) that allows for statistical testing of such differences. Note that finding consistent topographic differences between groups and/or conditions within the same microstate class weakens the notion that this is indeed always the same microstate class. In this case, we advise considering the shared variance of the involved group / or condition mean template maps, which indicates the shared variance in source space i.e. scalp topographies with high shared variances are assumed to have similar underlying generators compared to scalp topographies with lower shared variances may be generated by differing underlying generators. Also, such significant topographic differences give reason to explore these differences further using inverse solutions.

## Important Requirements and Choices in Microstate Analysis

### EEG Preprocessing Recommendations

As a precondition for microstate analysis, specific preprocessing steps should be conducted. These are described below.

#### Temporal and Spatial Filtering

EEG signals should be suitably band-pass filtered in time. Typical high-pass filter ranges from 1 and 2 Hz and the low-pass filter ranges from 20 and 40 Hz. This should eliminate large amplitudes, low frequency artifacts (sweating, skin potentials, etc.) and high frequency artifacts (muscle, line noise, etc.) while preserving the physiological brain signal. Note, however, that such high-pass filtering may be problematic for EEG recordings during sleep states. Depending on the data and common to other quantitative EEG methods, it may also be necessary to apply spatial filtering procedures like ICA-based removal of EOG artifacts (Jung et al. 2000). Such artifacts may otherwise later be falsely identified as microstates. It is worth noting here that the spatial fitting computations necessary in many steps of microstate analyses can be seen as the output of spatial filtering, which makes it obvious why insufficient or excessive spatial filtering during the preprocessing of the EEG signals will systematically distort the outcome of a subsequent microstate analysis. Finally, the EEG signals must be re-referenced to average reference after all bad channels have been removed or interpolated, which is equivalent to a spatial DC removal.

#### Sampling in Time and Space

The sampling rate must be compatible with the filtering rate to avoid aliasing. The electrode montage should cover the entire scalp uniformly and with a reasonable density. Zhang et al. (2021) does not recommend using less than the 10–20 system for a 4-class solution (Zhang et al. 2021). If more microstate classes are to be used or if inverse solutions are to be identified, a denser array is necessary (Michel and Brandeis 2009). The microstate toolbox is capable of handling different electrode montages within the same analysis.

#### Epoching Resting State Data and Artifact Detection

As in most other EEG analyses, the data should have been edited for artifacts before microstate analysis and meet commonly accepted standards in data quality. This typically entails that after eliminating time periods with artifacts, the EEG to be analyzed comes in several epochs of reasonably artefact free time periods. At the same time, the identification of microstates is compromised at the beginning and end of each EEG epoch because the onset or offset of the microstates at the epoch borders may have been truncated, and the true temporal extend of the microstate remains unknown. Therefore, EEG epochs should be as long as possible, avoiding the introduction of unnecessary epoch borders. Contrary to other EEG analytical approaches, such as ERP or FFT, EEG epoch sizes are not required to be constant.

### Choices in Clustering

#### Types of Clustering Approaches

For resting-state microstate analyses, we recommend using a modified k-means algorithm. The modification made to the algorithm ensures that maps of opposite polarity are considered equivalent, as described in Sect. 2. Because the outcome of the k-means algorithm depends on a randomly chosen starting condition, it has the problem that it may not always yield the globally optimal solution (i.e., the solution that explains the maximally possible amount of variance). Therefore, the toolbox offers the possibility to reinitialize with random new starting conditions and retain the overall best clustering solution. For exploratory data analysis, 5 restarts may be sufficient. For publications, at least 20 restarts are recommended. The toolbox also provides AAHC as an option which is yet to be tested and validated.

#### Number of Classes

The number of active classes in each dataset is an important parameter as it strongly affects the outcome but is difficult to determine as there may be no “true” number of microstates. A useful number of microstate classes should both capture the relevant details of the data and retain generalizability. Factors such as the contrasts of interests, the data quality, sample size, etc., may thus affect the suitability of a microstate solution with a given cluster number. We and others are in the process of implementing measures that determine the optimal number of cluster solutions in a data driven way and based on such objectives. The toolbox will be updated with these tools as they become available. In the meanwhile, we advise users to explore the effects of choosing different numbers of clusters on their experimental data sufficiently in order to achieve optimal understanding of the implications of their choices.

#### Selection of Data for Clustering/Downsampling

As outlined in the introduction, the microstate model assumes that there are periodic polarity reversals of the EEG field that represent the single global brain state. Therefore, for these moments of polarity reversal, the model cannot account for any data, and, in return, the measurements at these moments of polarity reversal cannot be accounted for by the microstate model (Michel and Koenig 2018). Two approaches have been used to overcome this problem during microstate template identification and backfitting these templates on raw data:

##### Clustering and Backfitting based on the Global Field Power Peak Maps

Backfitting the raw EEG data at GFP peaks makes use of the fact that under the given assumption that all the relevant processes for microstate analyses are explained by common in- and anti-phase oscillations in source and sensor space and assuming that there is some constant background noise, the microstate model has momentarily optimal signal to noise ratios at the assumed simultaneous peaks and troughs of these oscillations. These moments are therefore characterized by being momentary maxima of the global field power. A common solution to avoid the problem of accounting for the moments of polarity reversals is to select only maps at momentary peaks of the GFP for clustering, backfitting, and feature extraction, as these moments likely have the best signal-to-noise ratio. For the assignment of the remaining data, a nearest neighbor criterion can then be used which assigns the same label to the neighboring EEG samples as the GFP label it is most closely associated with. A limitation is that this approach may not account for certain microstates that may exist entirely between GFP peaks.

##### Clustering and Backfitting Across all Samples in the Raw Data and Applying Label Smoothing

Backfitting each discrete topographic map of the raw EEG and smoothing the labels makes use of the fact that for an EEG that is normally dominated by relatively slow oscillations, very short microstates are implausible, and, therefore, time periods assigned to the same microstate class that are only very brief are likely to be accounted for by noise. Such short periods inflate the number of transitions, but the clustering and backfitting procedures can include a penalty function for the number of state transitions (label smoothing). As a result, backfitting with such a penalty yields microstate assignments that explain minimally less variance while efficiently suppressing very short microstate assignments. When using this option, the recommended approach is to choose the parameters of the label smoothing such that brief transitory states at troughs of the GFP are reliably suppressed, while the remainder of the microstate assignments remains stable. For a mathematical definition of the smoothing parameters, see (Pascual-Marqui et al. 1995).

The toolbox includes the option to choose either approach. The more frequently used alternative is to extract microstate maps from GFP peaks only, which is also computationally cheaper. Therefore, we currently recommend extracting the individual template maps and backfitting the continuous EEG data at GFP peaks only. At the same time, we note that both the GFP peak selection and the label smoothing approach may somewhat bias the data toward overestimating microstate duration.

## Tutorial

### Installing the Toolbox and Dependencies

#### Toolbox Information

The EEGLAB toolbox for resting-state microstate analysis is a plugin for EEGLAB (Delorme and Makeig 2004), adding options for microstate analysis to the standard user interface of the program. The toolbox is open access for scientific purposes, with no guarantee for any obtained results. The toolbox requires MATLAB version 2022b or later and EEGLAB version 2021 or later. A comprehensive guide to EEGLAB for novice or general users is available here (https://eeglab.org/tutorials/). The toolbox was tested with MATLAB version 2022b and EEGLAB v2021.1. Note that in general, microstate networks may be investigated in averaged evoked potentials (obtained from task-related EEG) as well (e.g., Brandeis et al. 1995, 1998; Schiller et al. 2016). However, the microstate toolbox presented in this article was specifically developed for the analysis of resting-state EEG data and is not suited for averaged event-related potentials. We recommend using other software tools such as Ragu (Habermann et al. 2018; Koenig et al. 2011) or CARTOOL (Brunet et al. 2011) to analyze event-related data.

#### Download and Set Up the Toolbox

To run the microstate analysis, the *Statistics and Machine Learning Toolbox* and *Optimization Toolbox* are required. To download the toolbox, the EEGLAB plugin manager can be used using the menu item **File → Manage EEGLAB extensions**. For the toolbox to integrate into EEGLAB, its entire and unchanged file and folder structure must be located in the plugin-folder of the active EEGLAB installation, such that the file eegplugin_microstatelab.m is found in the subdirectory …/plugins/MICROSTATELAB in the EEGLAB directory.

#### Usage of the Toolbox

The toolbox is amenable to novice users and individuals with little MATLAB experience as well as seasoned MATLAB users. There are two ways of conducting resting-state microstate analyses using the toolbox. Conveniently, you can use the graphical user interface (GUI) of EEGLAB to access most functions. Alternatively, command line prompts can be used to call the functions, allowing that the entire analysis can be run as a script. An example script containing all the steps with recommended parameters is provided for users. This script contains information on the recommended folder structure organization that allows the import of individual files and structures them into groups based on the folder organization. The following sections provide details on how to analyze data using both the GUI and the command line approaches based on the analysis of a sample dataset.

**Fig. 2:**
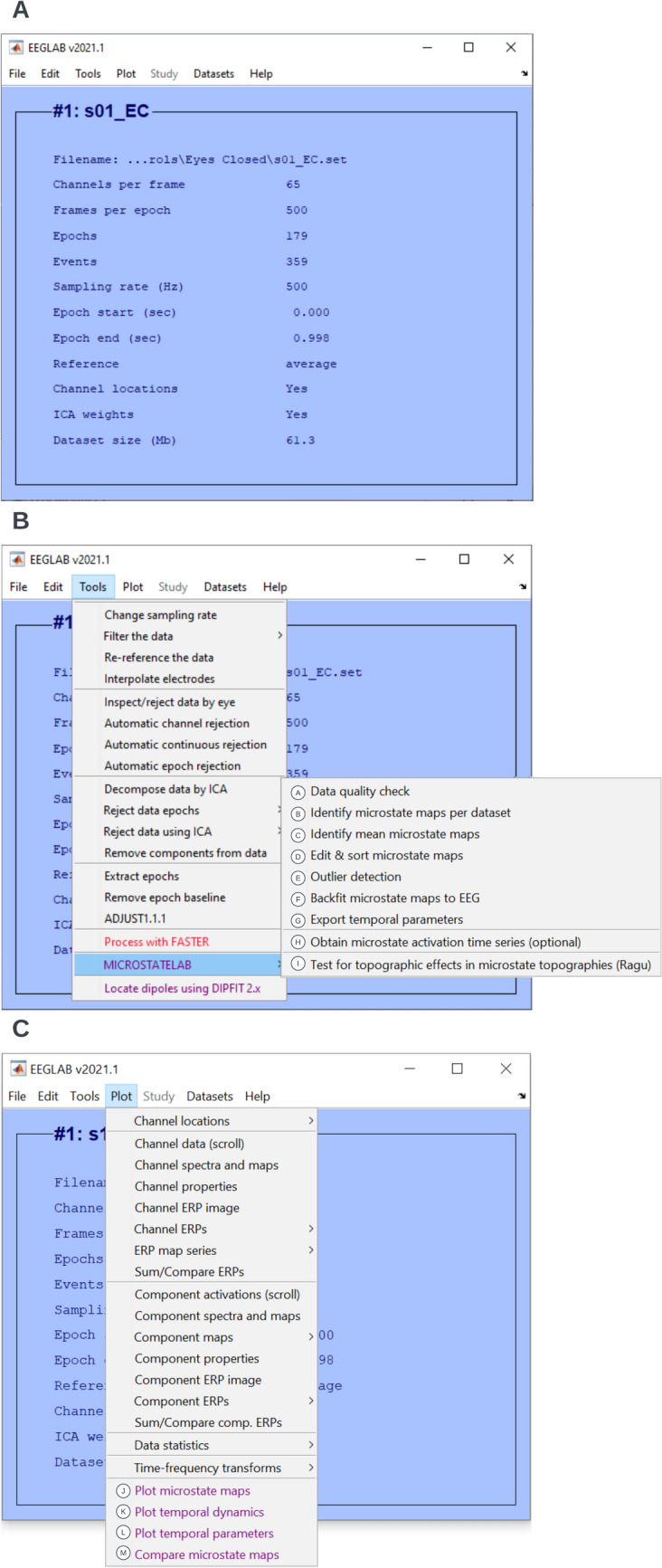
MICROSTATELAB GUI Overview. **A** Standard user interface of EEGLAB. Datasets can be accessed by using the menu option **Datasets**. **B** The microstate analysis steps can be accessed under the **Tools → MICROSTATELAB** option. **C** Microstate visualization options can be accessed by using the EEGLAB option **Plot**. The letters next to the microstate toolbox are referenced within the tutorial and are also cross-referenced in other GUI windows when applicable

The main microstate toolbox GUI functions are located in the EEGLAB menu **Tools**, and functions to visualize microstate maps that are in the menu **Plot** (Fig. 2).

Note that you can save the entire MATLAB workspace, including imported data and any microstate analysis steps by clicking **Home → Save Workspace** in MATLAB and choosing a directory. It is useful to do this from time to time, as some steps during microstate analysis can be quite time-consuming, depending on the sample size, sampling rate, and duration of EEG recordings. To load a saved workspace, click **Home → Open** and choose your saved workspace.

### Sample Dataset

**Fig. 3:**
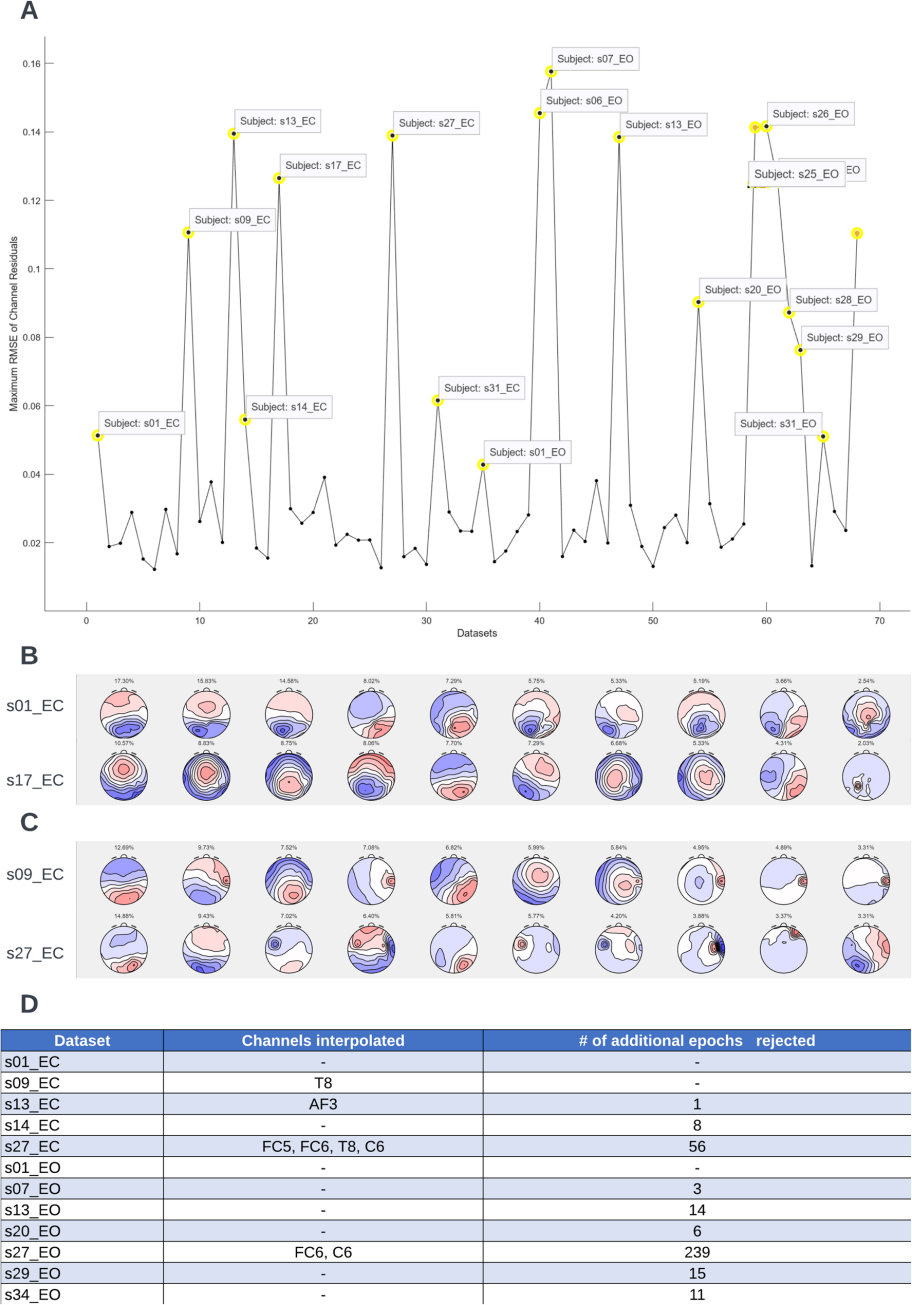
Data quality check. To check data quality, click ‘**Tools → MICROSTATELAB → Data quality check’** from the EEGLAB GUI. **A** Output of data quality check at a threshold of 0.04. The dataset marked for review, when selected, displays the topographies of the first 10 cluster solution maps. The data can be marked for exclusion. **B** and **C** Example topographies of datasets that are above the threshold of 0.04. **B** Example of datasets which contain very low amounts of residual artifacts that may not be addressed by further preprocessing. **C** Example datasets which contain a large amount of residual artifacts which may benefit from further preprocessing. **D** List of datasets with outlier maps and the additional reprocessing performed to address the bad topographies. Please note, no additional reprocessing was performed for datasets s01_EC and s01_EO as the maps marked as outliers are acceptable at the individual level

The sample datasets for this tutorial consists of 3 min of eyes open and eyes closed EEG data for 34 participants. These datasets are a subsample from the larger Dortmund Vital Study (for a detailed description of the study protocol, see (Gajewski et al. 2022)). The sample data and information about preprocessing can be found here: https://osf.io/yqt7k/. The **Microstate Analysis Sample Data → Pre-Outlier Detection** folder contains data prior to data quality evaluation with the toolbox’s outlier detection feature. The **Microstate Analysis Sample Data → Post-Outlier Detection** folder contains data that has undergone additional preprocessing (interpolation of bad channels and rejection of artifact-laden epochs after visual inspection by an expert rater, see Fig. 3D for details). The sample data are sorted into hierarchical folders and labeled according to the format described in Sect. 4.4.1.

### Exploratory Data Analysis Using the Graphical User Interface (GUI)

#### Evaluation of Data Quality

After your EEG data from the eyes closed and eyes open conditions from the **Sample data for microstate analysis—pre-outlier detection** folder are successfully loaded into EEGLAB and prior to starting microstate analysis, it is critical to identify datasets that may have residual artifacts in the EEG data. Large amounts of artifacts may result in atypical map topographies which affects all downstream analyses. This can be done by using the **Tools → MICROSTATELAB → Data quality check** option. An interactive GUI window is generated which allows users to set the threshold for detecting residual artifacts caused by bad channels (Fig. 3A). The **Auto select** option highlights datasets that contain residual artifacts above a selected threshold (here 0.04) which may affect the temporal parameters of that given dataset. Clicking on the highlighted points displays the name and topographies of the template maps for that dataset. These bad topographies can be addressed by further preprocessing. For large datasets, users can mark to **Keep** datasets that have minor topographical deviations or mark datasets to **Exclude** from the remainder of the analysis based on their judgment of the topographies if additional preprocessing is not an option. Figure 3B and C shows the outlier topographies obtained from the sample data. The numbers above the maps indicate the amount of data that a given map explains and the maps are ordered in decreasing order of explained variance. If the explained variance of an atypical map is very low and atypical maps do not appear within the maximum number of clusters the user intends on using for their analysis, no further preprocessing is needed (Fig. 3B). However, if the atypical maps explain large amounts of variance in the data, further preprocessing is recommended (Fig. 3C). The outliers in the sample dataset underwent additional preprocessing as indicated in Fig. 3D and were also made available.

#### Identify Individual Template Maps

**Fig. 4:**
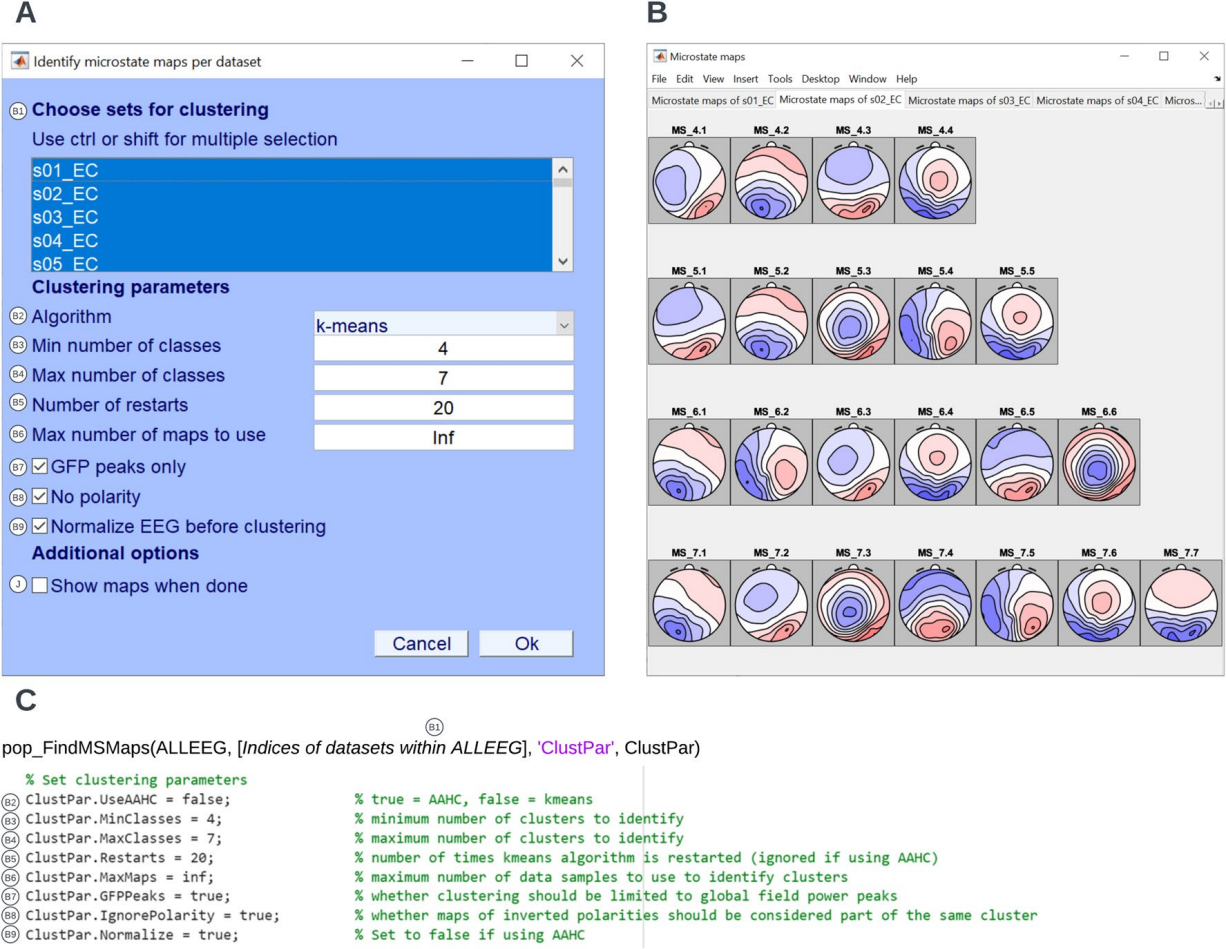
Identify individual template maps. To identify individual template maps, from the EEGLAB GUI click **Tools → MICROSTATELAB → Identify microstate maps per dataset**. **A** GUI window with clustering choices for identifying the individual template maps of each EEG dataset. Batch processing is available for this step by selecting all the desired datasets. Here, the k-means algorithm was chosen to identify 4–7 microstate class number solutions. The clustering was performed on all GFP peaks of normalized data with 20 restarts for any given dataset. Polarity was ignored during clustering. The maps can be viewed upon completion of clustering by checking box I. However, for a large number of datasets, it is recommended that this option is accessed through the **Plot** menu of EEGLAB. **B** Exemplary individual template maps across 4 through 7 cluster solutions for a dataset. The template maps of multiple datasets can be plotted in separate tabs by accessing it under the **Plot** menu of EEGLAB. At this stage, the microstates are not sorted as indicated by the gray background and the generic labeling for each class. **C** Excerpt of the command line function for identification of the individual template maps as used in the demo script provided. The various parameters corresponding to the GUI inputs are indicated

Once data quality evaluation has been performed and residual artifacts are addressed where appropriate, you can start your microstate analysis by identifying individual microstate maps. For the convenience of the users, this tutorial provides the cleaned data under the **Sample data for microstate analysis–post-outlier detection** folder. In EEGLAB, follow the path **Tools → MICROSTATELAB → Identify microstate maps per dataset**. The clustering parameters can be set in the popup window (Fig. 4A). Alternatively, you can set the parameters and perform the analysis by evaluating the following code in the toolbox-script (Fig. 4C). The unsorted, unlabeled individual microstate maps can be viewed by selecting **Plot → Plot microstate maps** (Fig. 4B).

#### Identify Mean and Grand Mean Maps

**Fig. 5:**
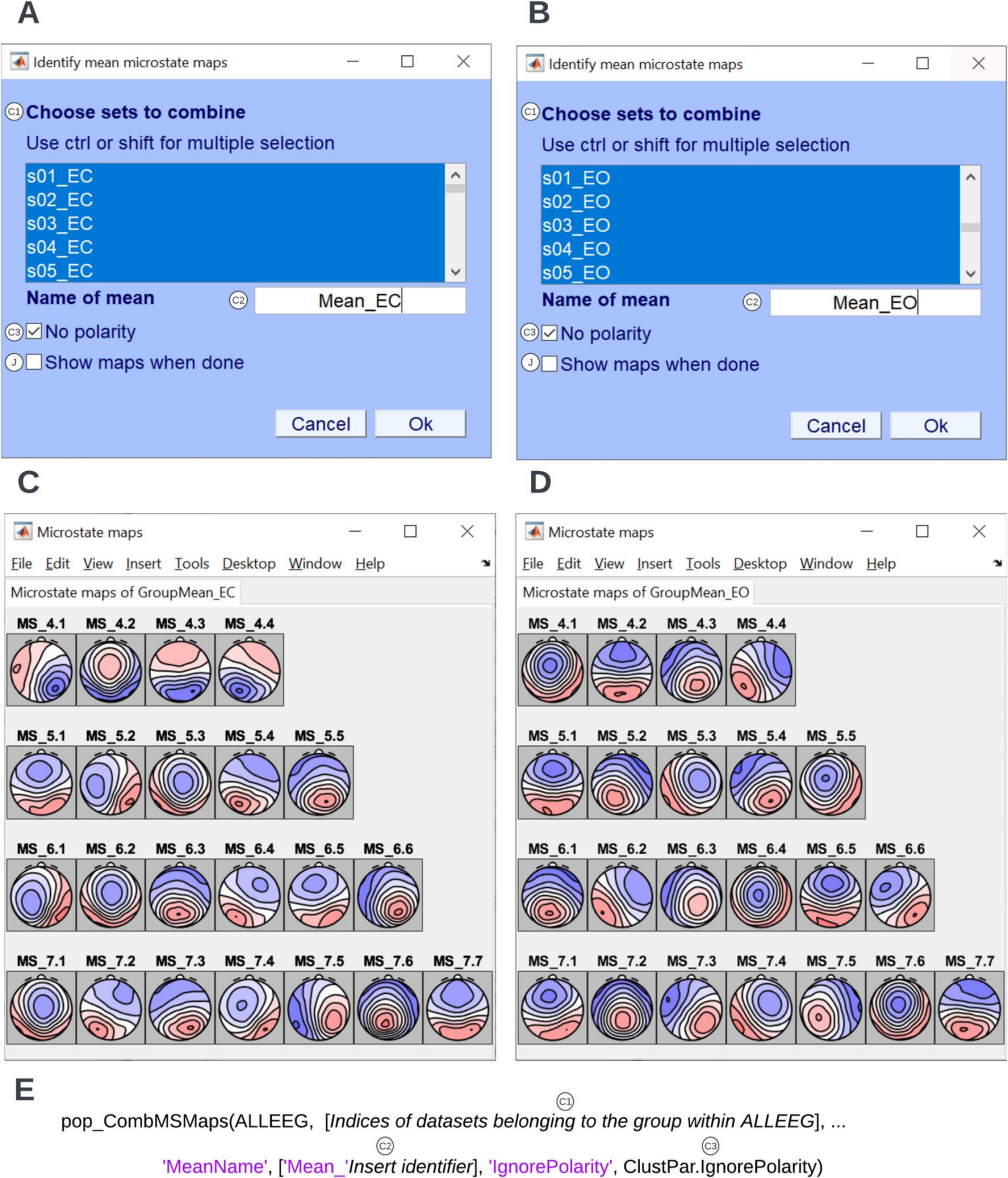
Identify mean maps across conditions. To identify the mean maps for the eyes closed and eyes open conditions, click **Tools → MICROSTATELAB → Identify mean microstate maps** from the EEGLAB GUI. **A** and **B** In the following window, the individual maps from the eyes-closed datasets and eyes-open datasets were selected. The respective group mean maps were labeled and clustered while ignoring polarity. **C** and **D** The topographies of the group-level template maps across 4 through 7 cluster solutions. The mean maps can be plotted in separate tabs by accessing it under the **Plot** menu of EEGLAB. These maps are also not yet sorted as indicated by the gray background and the generic labeling. **E** Excerpt of the command line function for identification of the mean maps as used in the demo script provided. The various parameters corresponding to the GUI inputs are indicated

**Fig. 6:**
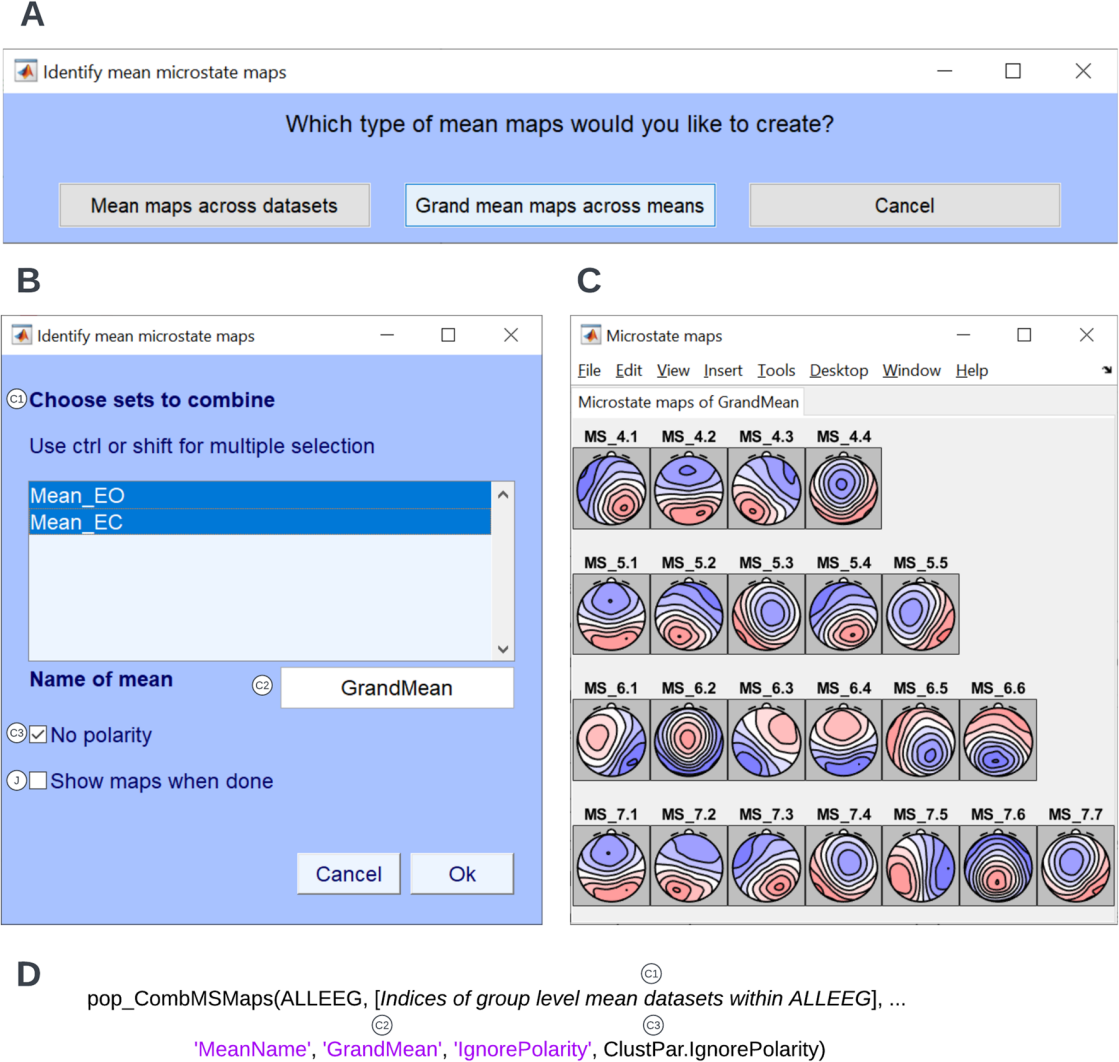
Identify grand mean maps. To identify the group-level template maps for the eyes closed and eyes open conditions, click **Tools → MICROSTATELAB → Identify mean microstate maps** from the EEGLAB GUI. **A** Here, the option **Grand mean maps across means** was chosen. **B** The mean maps for eyes closed and eyes open conditions were selected and the grand mean maps are computed while ignoring polarity. **C** The topographies of the grand mean maps across 4 through 7 cluster solutions. The microstate classes are not yet sorted as indicated by the gray background and the generic labeling. **D** Excerpt of the command line function for identification of the grand mean microstate maps as used in the demo script provided. The various parameters corresponding to the GUI inputs are indicated

This toolbox allows for the identification of the group- or condition-level mean microstate maps as well as the grand mean microstate maps. A stepwise computation of the grand mean microstate maps after computing intermediate group- or condition-level mean microstate maps may be useful if one wants to equally weight experimental groups of different sizes. To identify mean maps, follow the path **Tools → MICROSTATELAB → Identify mean microstate maps**. Select the members belonging to the group or condition and provide the group name Fig. 5A, B). This process is repeated across all groups and/or conditions. If two or more mean maps are present for groups and/or conditions, a grand mean must be computed. To identify the grand mean microstate maps, follow the path, **Tools → MICROSTATELAB → Identify mean microstate maps → Grand mean maps across means** (Fig. 6A, B). The unsorted, unlabeled group or condition-level means and grand mean microstate maps can be viewed by selecting **Plot → Plot microstate maps** (Figs. 5C, D and 6C). These operations can also be performed using the command line scripts described in Figs. 5E and 6D respectively.

#### Sort Grand Mean, Group-Level and Individual Template Maps

For the convenience of the user, this toolbox allows the sorting and labeling of the grand mean microstate maps according to the overall highest spatial correlation with published template maps and also provides an option to manually sort and label the maps. By default, the toolbox contains the four to seven prototypical microstate classes identified by Koenig et al. (2002) and the seven microstate maps identified by Custo et al. (2017), enabling the sorting of individual microstate maps according to these normative template maps. Note that other previously published templates may be used as well (e.g., with a different cluster number). These maps are stored in a dedicated folder in the microstate plugin and can be imported to be used as the published template used to sort the grand mean.

**Fig. 7:**
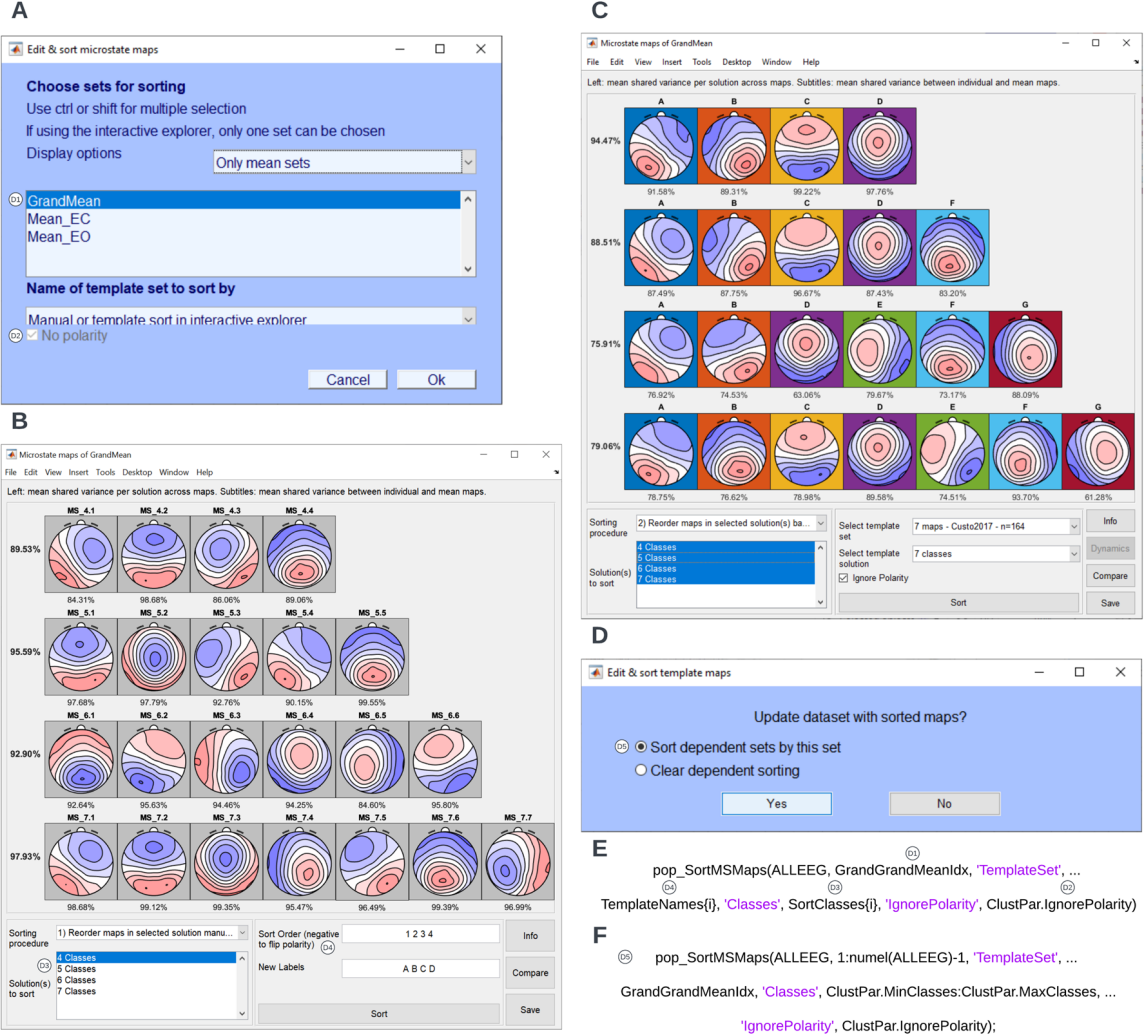
Sort grand mean template maps. To sort the mean template maps, click **Tools → MICROSTATELAB → Edit & sort template maps** from the EEGLAB GUI. **A** Here, select the **GrandMean** dataset and select the **Manual or template sort in interactive explorer** option. **B** The interactive sorting window with the grand mean template maps. For the sorting procedure, select **2) Reorder maps in selected solution(s) based on template set**. Select all the classes for the solutions to sort. Select the published template map. Here, we chose the 7-class solution by Custo et al. (2017) as the published template to sort by. Note that the sorting of template maps can also be done manually. **C** The sorted and labeled grand mean template maps across 4 through 7 cluster solutions. **D** The group-level template maps and the individual template maps and sorted and relabel across 4 through 7 solutions for appropriate comparison by selecting the **Sort dependent sets by this set** option. **E** and **F** Excerpt of the command line function for sorting of the grand mean template maps and the dependent individual and group mean template maps respectively as used in the demo script provided. The various parameters corresponding to the GUI inputs are indicated

Sorting can be performed by clicking **Tools → MICROSTATELAB → Edit & sort microstate maps** and selecting the grand mean map to be sorted (Fig. 7A). The unsorted grand mean maps and the various approaches for sorting the grand mean are displayed in a new window (Fig. 7B). In this tutorial we will demonstrate the recommended approach of first sorting the 7-class solution of the grand mean by the 2017 Custo maps and then sorting the lower number of clusters based on the 7 classes of the sorted grand mean. This approach yields the greatest within class spatial correlations across different cluster number solutions (Fig. 7C) and therefore, maximizes the comparability of results obtained with different cluster numbers.

Upon saving the sorting, the toolbox provides the option to **Sort all dependent sets by this set** which sorts all group-level and individual template maps by the grand mean which serves as the template map sorting (Fig. 7D). This sorting step of these individual microstate maps is crucial when they are later used to quantify individual EEG microstate dynamics or when microstate template maps are compared between group or conditions (see below), as only after this step, common labels of these individual template maps can be assumed to refer to similar topographies. This is essential when comparing maps (Sect. 4.3.5 and 4.3.9), when detecting outlier microstate templates (Sect. 4.3.6), and when backfitting and quantifying microstates (Sects. 4.3.7 and 4.3.8), as the results of these analysis will be meaningless otherwise.

The sorting for the grand mean dataset can be revisited by the **Tools → MICROSTATELAB → Edit & sort template maps**, selecting the grand mean template and choosing the **Manual or template sort in interactive explorer** option.

#### Compare Maps

**Fig. 8:**
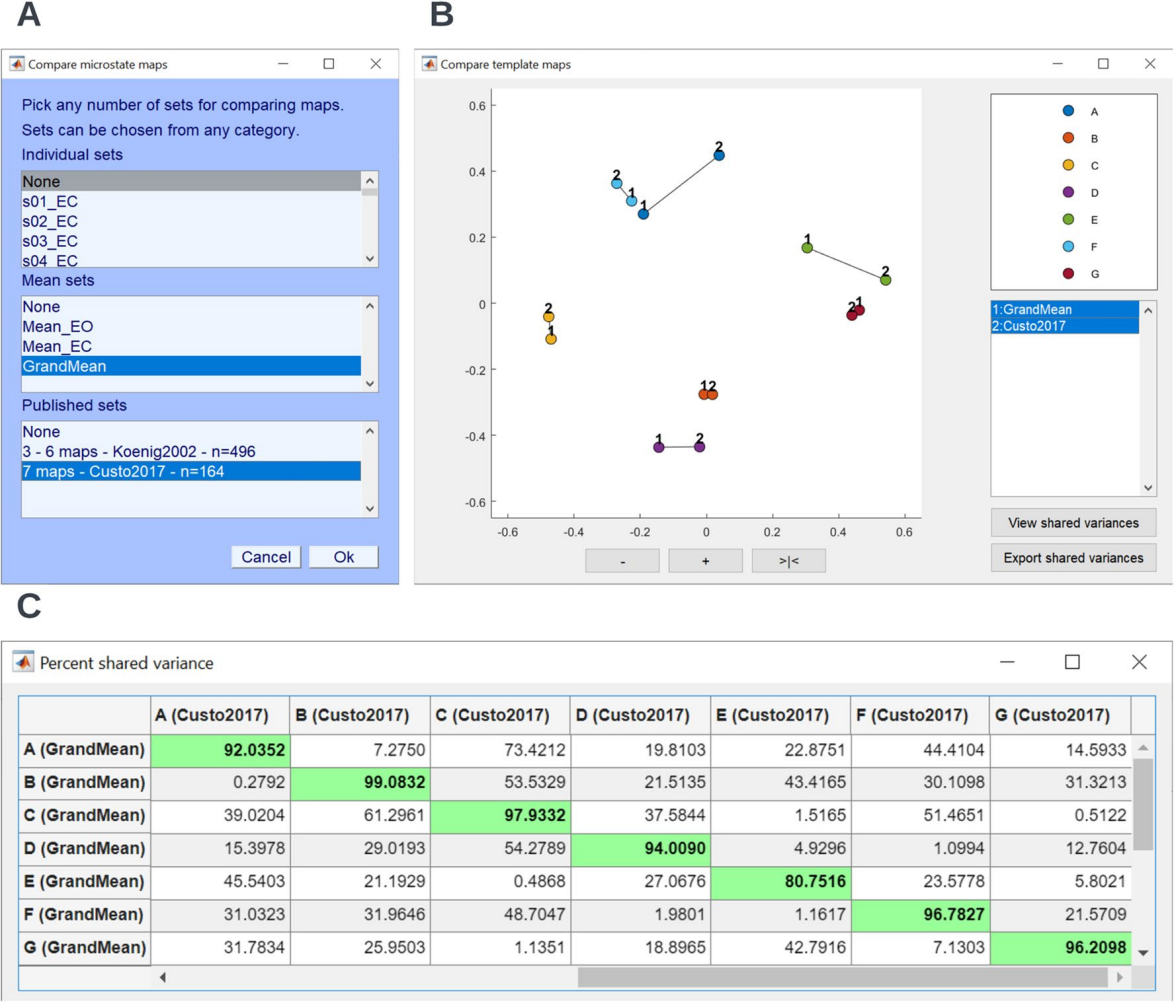
Comparing the topographical similarity of the grand mean template maps with published template maps. To compare the similarity across published template maps and the grand mean template maps obtained from the sample data, the option **Compare microstate maps** under the EEGLAB **Plot** menu can be used. **A** GUI window for selection of datasets to be compared. Any number of datasets can be selected in the three categories. Here, no individual template maps were selected and only the new grand mean template maps were selected among the mean sets and the maps by Custo et al. (2017) were selected among the published sets. **B** Multidimensional scaling output of the correlation matrix showing the relationship between the grand mean template maps and the 2017 Custo maps. The graph represents each of the selected template maps by a two-dimensional point that is labelled using the color and number scheme on the right side. The relative positions of these points are chosen such that the distances between the points maximally correspond to the topographic similarities among the maps, as they are tabulated in part C. Points that are close together therefore represent spatially similar maps, and points far apart represent spatially different maps. The graph therefore allows for an intuitive visualization of the topographic consistency of microstate templates across number of clusters, conditions, groups or studies. **C** Shared variances between the new grand mean template maps and the maps by Custo et al (2017)

Once the sorting and labeling is obtained, the topographical similarity of classes across the various cluster number solutions can be evaluated using the path **Plot → Compare microstate maps**. The topographical similarities within datasets and across datasets can be evaluated for individual datasets or across multiple datasets respectively. Here, we demonstrate this feature by comparing the grand mean maps of our sample data with the published maps by Custo et al. (2017 maps (Fig. 8A). The interactive GUI popup shows the topographical similarity of the grand mean template maps with the maps by (Custo et al. 2017) in an MDS (Fig. 8B). The shared variances can be viewed or exported for further analyses using the **View shared variances** and/or **Export shared variances** buttons in the GUI (Fig. 8C). The shared variances between the grand mean maps from our dataset with those from (Custo et al. 2017) are mostly high (80–99%). These similarities in scalp topographies can be interpreted as the similarities in the spatial distribution and orientation of the EEG sources i.e., the brain networks generating these scalp fields.

#### Outlier Detection

**Fig. 9:**
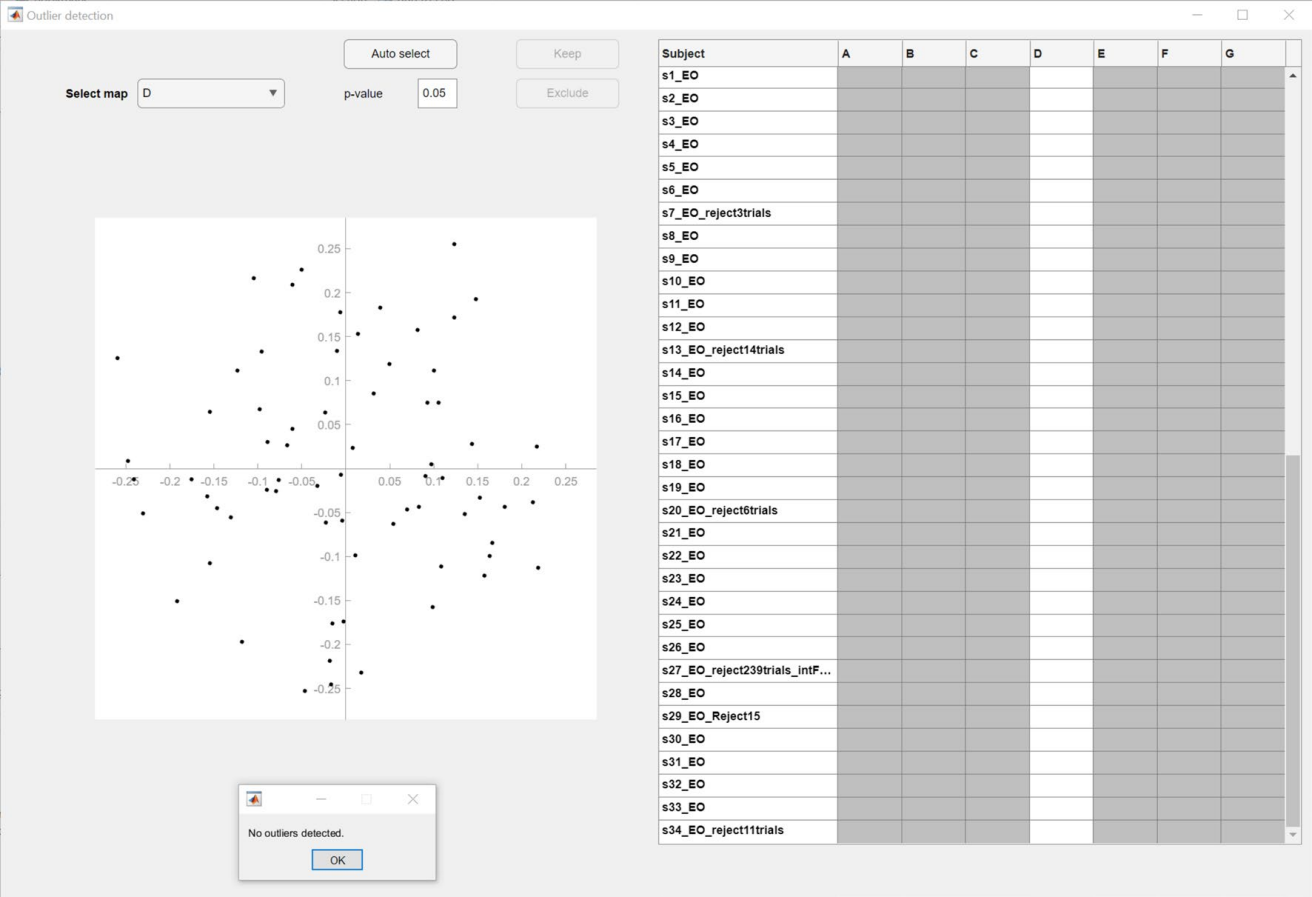
Detection of outlier maps. Once all data has been reprocessed and the above steps are repeated with the reprocessed data, outlier detection for bad topographies can be performed by clicking **Tools → MICROSTATELAB → Outlier detection** from the EEGLAB GUI. With a p value of 0.05, no bad topographies were detected across the 7 classes in any individual

Topographical outliers of individual EEG microstate maps can be detected by selecting the option **Tools → MICROSTATELAB → Outlier Detection**. If multiple cluster solutions are available, the user must perform this step across all the cluster solutions if the analyses are exploratory or if a cluster number is chosen a priori, then outlier detection should be performed on that cluster number. The resulting window displays the coordinates generated by the multidimensional scaling (MDS) algorithm of each individual dataset for the selected classes (Fig. 9). For more details on the MDS algorithm, please refer to (Habermann et al. 2018). Selecting the points representing individual datasets generates the corresponding map of the selected class for review. Selected datasets can be excluded by manual inspection in a sequential manner, or using the auto-select option, which identifies outliers, based on the Mahalanobis distance amongst the datasets that indicates the datasets least likely to be part of a normal distribution comprised of those datasets. Outlier datasets may necessitate further preprocessing. It is recommended that users inspect the topographies of all individual and group-level template maps by using the **Plot → Plot microstate maps** option to ensure sound topographies across their own data. Our data did not generate topographic outliers. However, if users encounter topographic outliers in their data that are not addressed with further preprocessing, it is recommended that the users exclude these datasets and repeat steps outlined in Sects. 4.3.3 onwards to generate new mean and grand mean maps to account for the excluded datasets.

#### Backfit and Quantify Microstate Dynamics

**Fig. 10:**
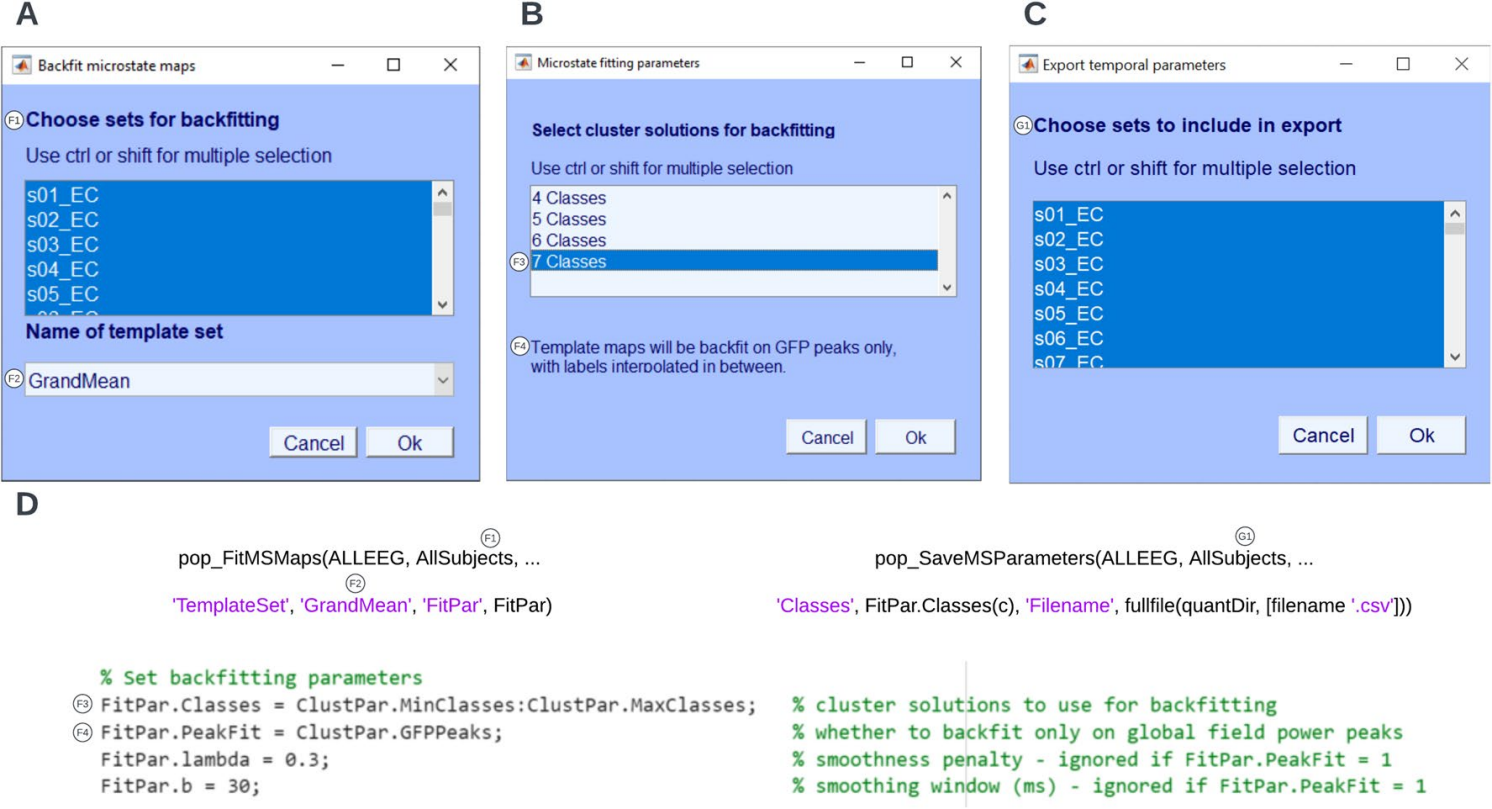
Backfitting and exporting quantification of temporal parameters. The data can be backfit using the menu option **Tools → MICROSTATELAB → Backfit microstate maps to EEG**. **A** GUI window to select the individual datasets to be backfit and the template map to be used for backfitting. Here, the grand mean microstate maps are chosen. **B** Selection of the cluster solutions to be used for backfitting. One or more options can be chosen. **C** Once backfitting is complete, the temporal parameters of the individual subjects can be exported by selecting the datasets in the GUI window which can be accessed by clicking **Tools → MICROSTATELAB → Export temporal parameters**. Please note, temporal parameters can only be extracted for the cluster numbers that underwent backfitting in the previous step. **D** Command line prompts for backfitting and exporting temporal parameters

**Fig. 11:**
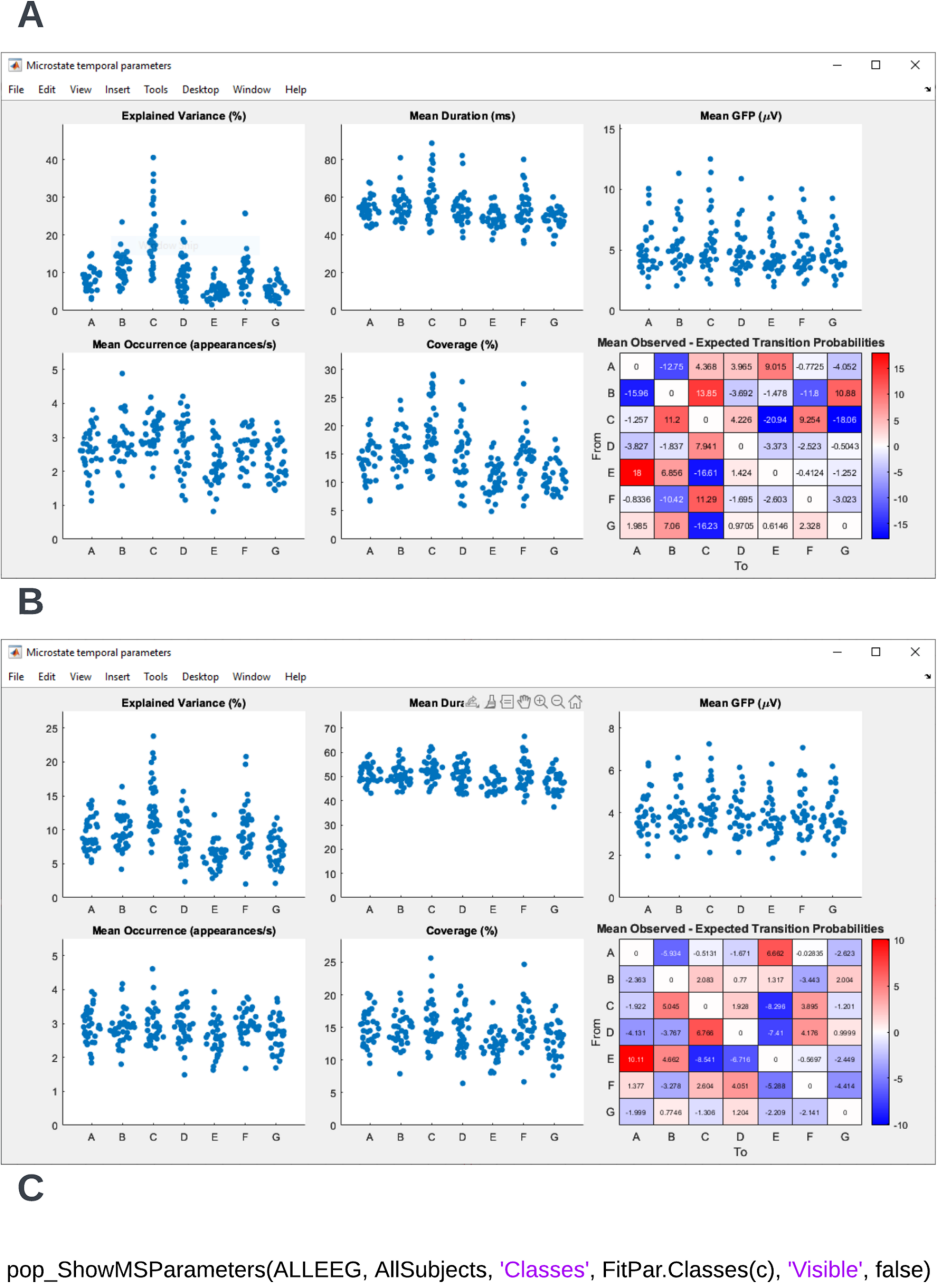
Visualization of the temporal parameters of EEG microstates. The temporal parameters of the individual subjects can be visualized by selecting the **Plot → Plot temporal parameters**. **A** Temporal parameters of the eyes closed datasets. **B** Temporal parameters of the eyes open datasets. **C** Command line prompt for plotting temporal parameters

**Fig. 12:**
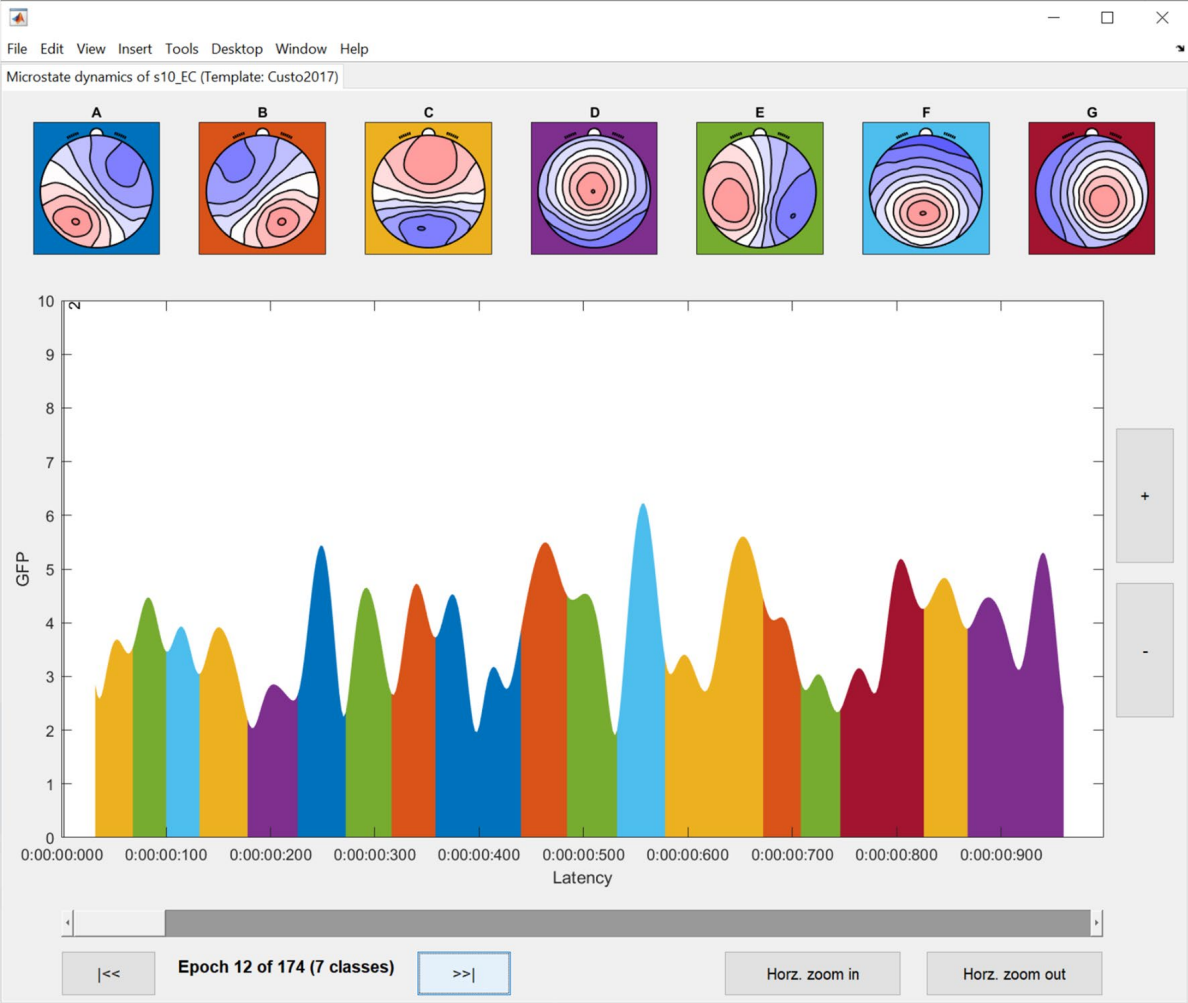
Visualization of the temporal dynamics of EEG microstates at an individual subject level. To view the subject level microstate dynamics, the **Plot temporal dynamics** under the **Plot** menu of EEGLAB can be selected. The dynamics can be visualized on an epoch-by-epoch basis

To extract the temporal dynamics of microstates, the raw EEG of individual datasets can be re-expressed as a sequence of microstate classes by following the path **Tools → MICROSTATELAB → Backfit microstate maps to EEG**. Here, we recommend choosing the grand mean microstate maps as the template for backfitting as explained in Section 3.3 above (Fig. 10A). One or more cluster number solutions can be chosen (Fig. 10B) for the desired datasets (Fig. 10C). The resulting output of the summarized temporal parameters can be visualized by selecting **Plot → Plot temporal parameters** for each cluster number solution of interest for the eyes closed (Fig. 11A) and eyes open (Fig. 11B) conditions. The summarized data can be exported for statistical testing by using the option **Tools → MICROSTATELAB → Export temporal parameters**. The resulting file is saved in the location of the user’s preference and contains the temporal parameters described in Table 1. The toolbox the option to export the results in different output formats which can directly be imported into statistical applications such as SPSS or R. The timeseries of microstate classes for individual datasets can be examined by choosing the option **Plot → Plot microstate dynamics** (Fig. 12). Note here that the exported.

#### Export of Backfitting Time-Series

**Fig. 13:**
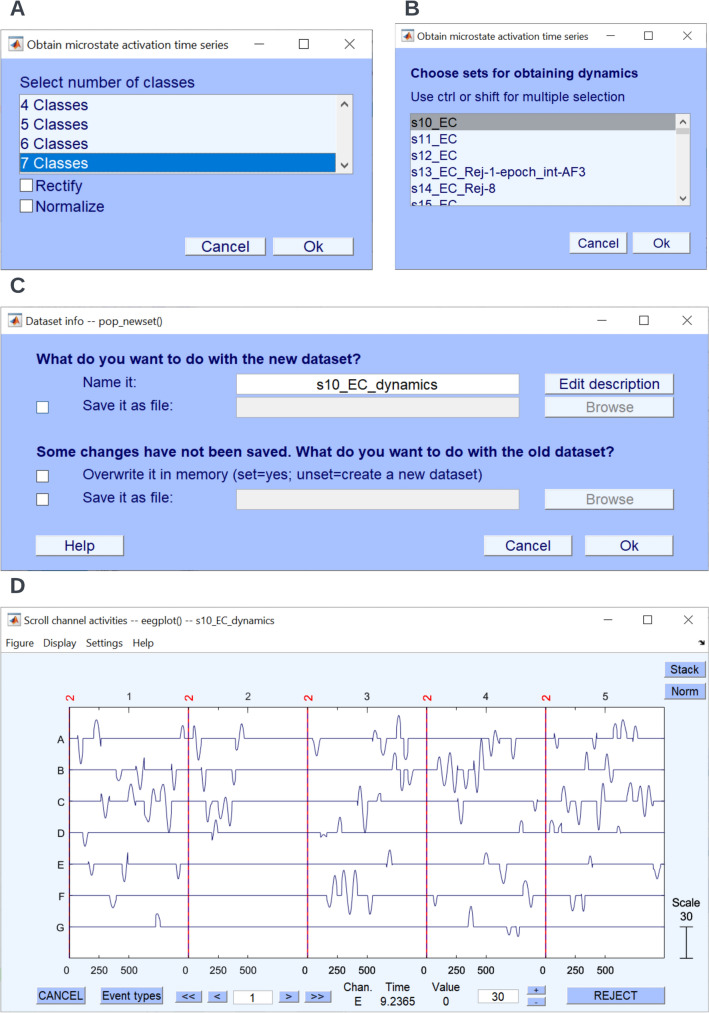
Timeseries of the fit of individual microstate classes with the EEG data. The time series of spatial correlations of individual microstate classes can be obtained using the menu option **Tools → MICROSTATELAB → Obtain microstate activation time series (Optional)**. **A** Select the number of classes. **B** Select the dataset of interest. **C** Save the newly generated dataset with the microstate time series activations. **D** View the microstate time series activations by selecting **Plot → Channel data (scroll)**

For some research questions, it may be interesting to relate the ongoing microstate dynamics to external events that were marked in the EEG. For this purpose, it is possible to produce new EEGLAB datasets that contain time-series of microstate assignments. These time-series are stored like ordinary EEG channels, where each channel represents one microstate class, allowing to use all the standard tools of EEGLAB (like epoching or averaging) to work with such data. Users can produce such datasets using the option **Tools → MICROSTATELAB → Obtain microstate activation time series (Optional)**. Select the number of classes (Fig. 13A) and the dataset of interest (Fig. 13B). This generates a new dataset with the suffix ‘_dynamics’ (Fig. 13). By default, the output for channel X is the dot-product of the momentary EEG topography with the microstate template map X for periods where the EEG was assigned to class X, and zero for all other periods. In addition, users have the option to rectify and/or normalize the data, producing a binary on–off pattern. Averaging such binary time-series of microstates in reference to a series of events thus yields the probability of observing particular microstate classes in reference to the events (see e.g., (Mikutta et al. 2023; Müller et al. 2005), for examples). The timeseries of microstate assignment can be plotted by using the EEGLAB function **Plot → Channel data (scroll)** for the newly created dynamics dataset (Fig. 13D).

#### Export to Ragu for TANOVA

Although the group-level clustering procedure aims at maximizing the commonality of each microstate class across subjects, it might be that there are systematic differences in the spatial distribution of one or more microstate classes between certain subgroups of your sample. As differences in spatial distributions may indicate differences in underlying neural sources, you might want to test for statistically significant differences between microstate maps. To do so, you can export individual microstate maps to functions of the software Ragu (Habermann et al. 2018; Koenig et al. 2011), which allows you to statistically test for differences between microstate maps by using topographic analysis of variance (TANOVA), post-hoc tests, and t-maps (Habermann et al. 2018). Please note, no additional toolboxes are necessary to perform the TANOVA as these functions are included within MICROSTATELAB.

**Fig. 14:**
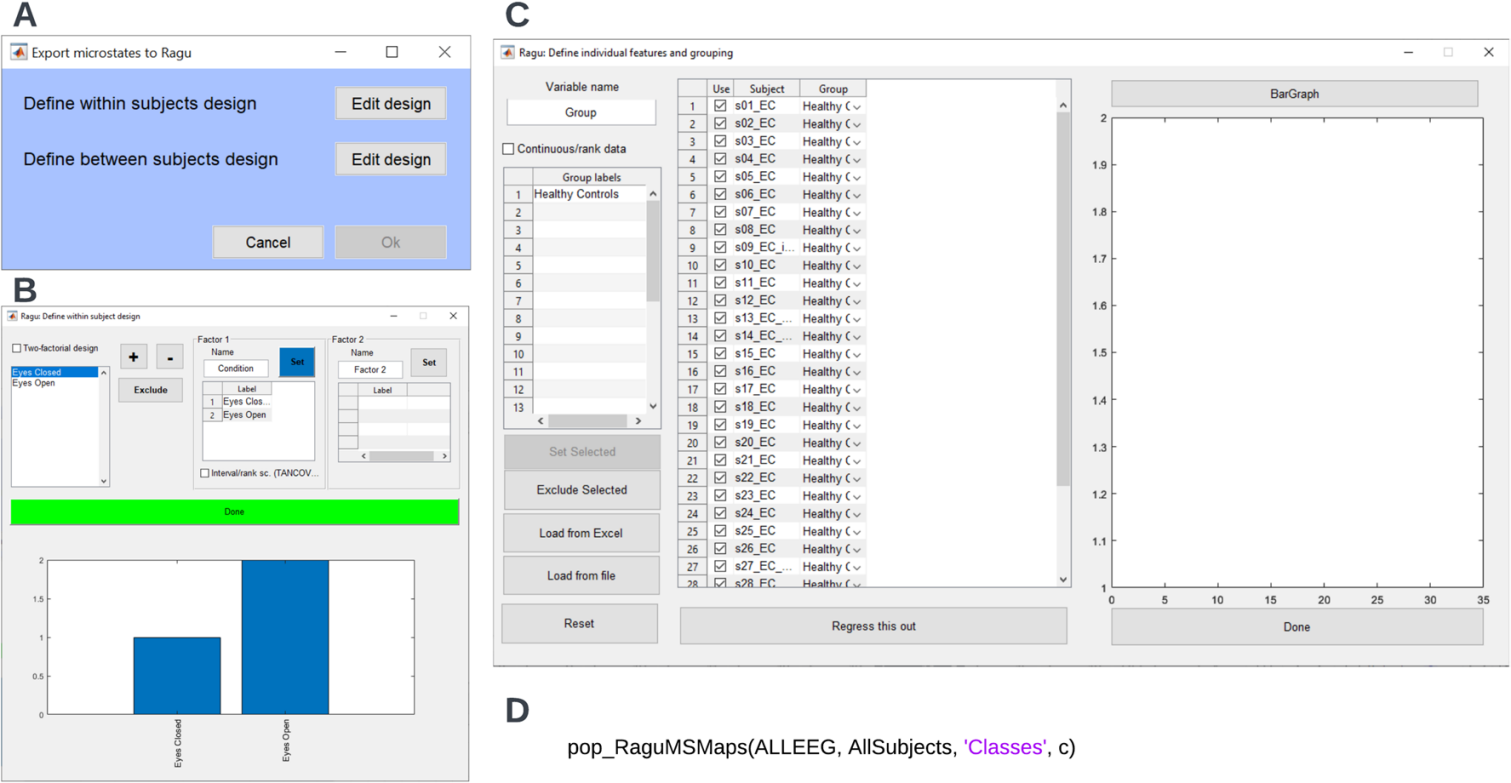
TANOVA set up. The data can be exported to Ragu for comparison of topographical differences with TANOVA by selecting **Tools → MICROSTATELAB → Test for topographic effects in microstate topographies (Ragu). A** Menu to edit the within- and between-subject design. **B** Interactive window to set the within-subject design. The sample data has only one factor—condition. The assignment of the condition to the different levels is shown. **C** Interactive window to set the between-subjects design. The sample data has only one group—healthy controls. **D** Command line prompt for exporting data to Ragu

**Fig. 15:**
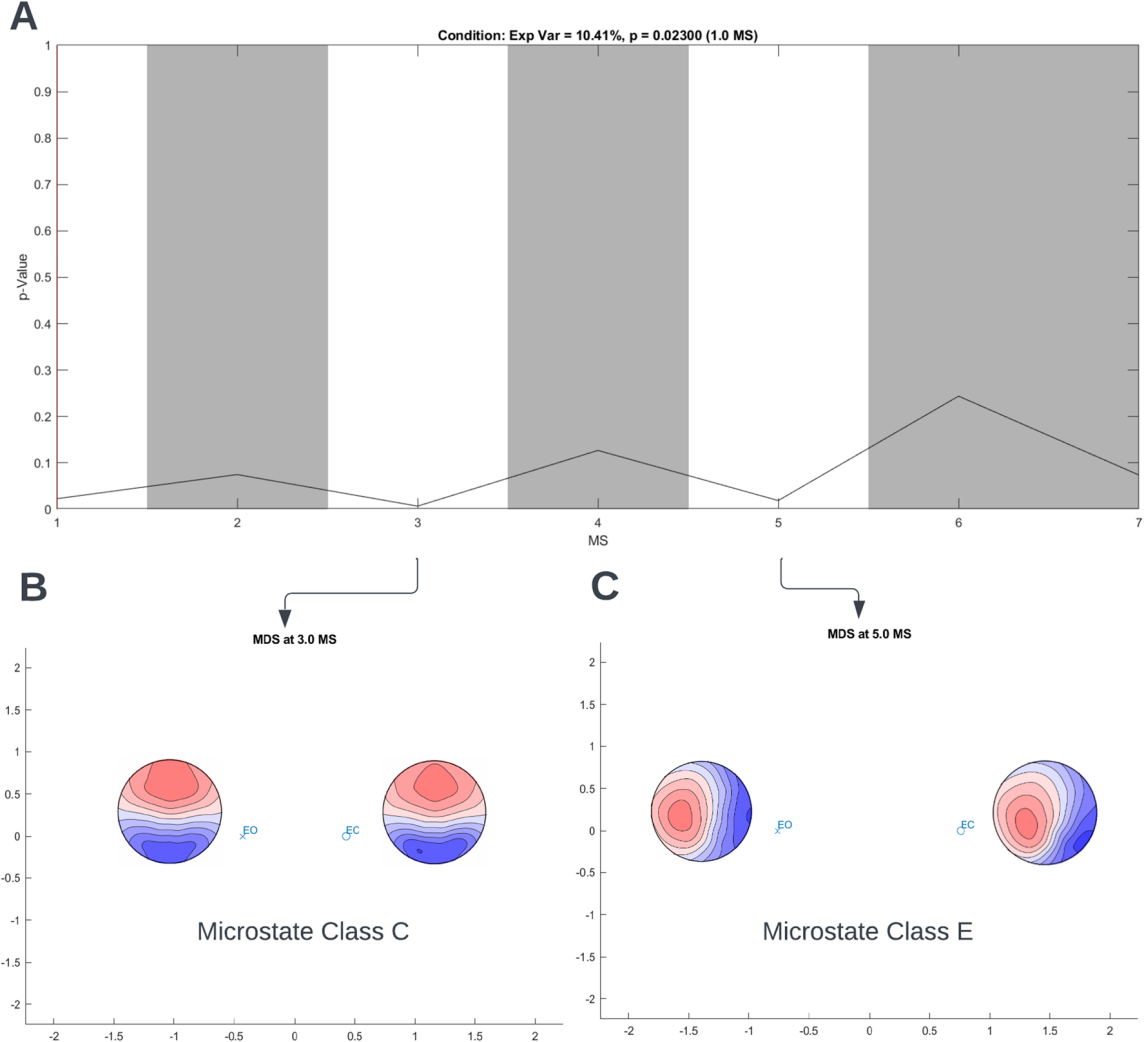
Example TANOVA results for the sample data. **A** The p values (y-axis) for the comparison between the mean maps for the eyes closed and eyes open condition for each microstate class (x-axis) labeled 1–7 which corresponds to classes **A**–**G**. The white areas indicate maps with significant microstate topography differences between the eyes closed and eyes open conditions, i.e., maps of classes **A**, **C**, and **E**. **B** and **C** State-space representation of maps **C** and **E** respectively for eyes closed and eyes open conditions

To export individual microstate maps for the analysis in Ragu, choose the function **Tools → MICROSTATELAB → Test for topographic effects in microstate topographies (Ragu)**. Using the popup window (Fig. 14A), edit the within (Fig. 14B) and between subjects design (Fig. 14C). For detailed information on conducting the TANOVA, please refer to Habermann et al. (2018). The comparison between the eyes closed and eyes open microstate maps is displayed in Fig. 15A. The state-space representation of the maps for Class C and E, that are significantly different, are displayed in Fig. 15B and C respectively. Given the high degree of spatial correlation between the grand mean maps and the maps by Custo et al. 2017, the results of the TANOVA indicate differences in the generators of the maps representing the default mode network between the eyes closed and eyes open conditions.

### Batch Processing Data Using Toolbox Script

A sample script, **MicrostateAnalysisDemo.m**, has been provided with the toolbox to aid novice and experienced users with batch processing large datasets in a standardized manner and to generate reproducible results across runs. The input for the script and the output generated are described below.

#### Structuring the Preprocessed Data for Import

**Fig. 16:**
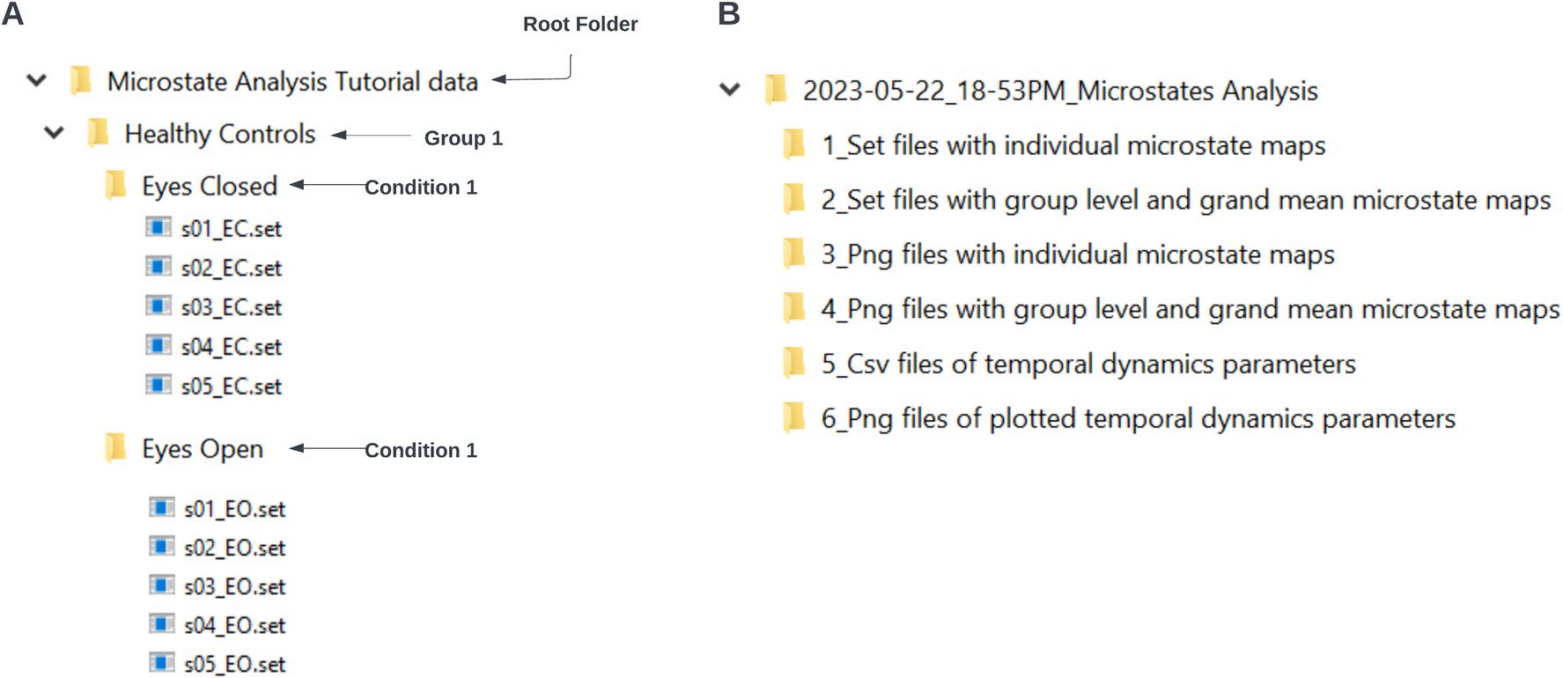
Input (**A**) and output (**B**) folder structure of the demo script provided with this toolbox

The script requires the input folder to follow a strict structure so that the group and condition information can be read correctly. The root folder should contain the group level folders which should then contain the condition level folders (Fig. 16A). The condition level folders should have the datasets of the individual subjects. It is crucial that the individual datasets are labeled with a unique identifier of the subject that is consistent across conditions for that subject, as well as the experimental conditions. This format is particularly important for conducting the TANOVA analysis in the final step. Note that the root folder, the group-level, and the condition-level folders should not contain any other files or folders. The script loads individual subject files that are in the EEGLAB.set format. This can be easily modified using EEGLAB import functions to be suitable for other file formats. Upon launching the script, the root folder containing the input data in the group and condition hierarchy can be selected from the interactive GUI menu.

#### Setting Key Analytical Parameters

The GUI inputs described in Sect. 4.3 can be set in Part 1 of the **MicrostateAnalysisDemo.m** script. These include selection of the clustering parameters for the identification of individual template maps (% Set clustering parameters), selection of parameters for backfitting (% Set backfitting parameters), and the template maps to be used for sorting the grand mean template maps (% Template sorting). The parameters corresponding to the menu options in the GUI are indicated by the letter insets.

#### Output of Standardized Script

The data generated by running **MicrostateAnalysisDemo.m** script are saved in a standardized format in the location chosen by the user in the interactive GUI (Fig. 16B) or defined in Part 1 of the script. All output folders contain a timestamp prefix for documentation purposes. A copy of the script used to generate the output is saved for documentation of the parameters used for that specific run. The subject files with the individual microstate template maps are saved in the **1_Set files with individual microstate maps** folder, the group-level and grand mean template maps are stored in the **2_Set files with group level and grand mean microstate maps** folder, all of which can be readily imported into EEGLAB. These datasets contain microstate maps that have been sorted according to the parameters used in the script and can be visualized using the GUI options described in Sect. 4.3 upon import. For ease of visual inspection of large datasets, the script saves the figures of the individual, group-level, and grand mean template maps in the folders **3_Png files with individual microstate maps** and **4_Png files with group level and grand mean microstate maps** respectively. These figures can serve as a guide for manual detection of outliers caused by noisy channels or residual artifacts. Please note, at this time, the script does not include the option for outlier detection. However, the data can be imported into EEGLAB and outlier detection can be performed using the GUI options described in Sect. 4.3.4. The temporal parameters are extracted using the individual template maps, the grand mean template maps and the published template maps and the output is saved in.csv format in the **5_Csv files of template dynamics parameters** and the visualizations of the same are saved under the **6_Png files of plotted temporal dynamics parameters**. All of these options can be easily modified by updating Part 9 of the demo script.

## Discussion

The present paper presents a tutorial for the second version of the *EEGLAB toolbox for resting-state microstate analysis* or *MICROSTATELAB*. The toolbox and tutorial assemble our current best understanding and recommendation of how to conduct resting state EEG microstate analysis. For standard applications, the toolbox can be accessed through a comprehensive and efficient GUI. The GUI is structured to guide the user step by step through a complete microstate analysis and contains some guardrails and default choices that protect against common pitfalls. In addition, the GUI comes with a series of comprehensive visualizations of intermediate results, making the necessary data quality checks an integral part of the analysis. The tutorial parallels each of these analysis steps and explains and justifies the most important choices for each step. The tutorial and the toolbox should thus enable users with a good general understanding of EEG to understand the basic rationales and necessary choices behind each analysis step and conduct a state-of-the-art microstate analysis.

Beyond implementing the current practice, the standard GUI based analysis pipeline comes with some easily accessible features that are not yet standard, but that we deem good practice for future microstate studies. Namely, users have efficient, interactive, and visually informative means to detect outliers in microstate maps, which should help improve the data quality also in larger datasets. This is important because so far, none of the existing automatic resting-state EEG preprocessing pipelines are fully reliable when it comes to making raw-EEG data fit for microstate analysis (Nagabhushan Kalburgi et al., this issue). Second, the toolbox comes with a broad range of options to sort the obtained microstate template maps both across solutions with different clusters, and in reference to previously published template maps, which dovetails with the attempt to develop tools to objectively integrate across different resting-state EEG microstate studies (Koenig et al., this issue). Finally, the toolbox allows to quantify and statistically test for spatial differences between microstate template maps using TANOVAs, allowing the user to only assess and test differences in microstate features, but also in microstate topography. This is relevant, because many studies falsely assume that a high spatial similarity between e.g., group mean microstate template maps is a reason to believe that there is no systematic topographic difference between these maps.

Beyond the extended standard case, the toolbox allows manipulating a large range of analysis parameters using the MATLAB command-line and scripting interface. Expert users can thus easily use the toolbox to step into less conventional questions, such as event-related changes in continuous EEG microstates, pattern recognition in microstate sequences or other, and add further methods to the toolbox. As the toolbox is open-source, users proficient in MATLAB may also get an in-depth understanding of the methodology by examining the code and providing corrections and improvements for future releases if necessary.

Contrary to other toolboxes, the toolbox does not yet contain an automatic procedure to select the number of classes. We have extensively tested many of the proposed criteria and found the outcome controversial so far. At the same time, we think that cluster structure in brain functional data may not be limited to a single scale (Van De Ville et al. 2010), which may further complicate the issue finding the ‘right’ number of classes, and it seems problematic to us if the conclusions drawn from a comprehensive EEG microstate analysis heavily depend on a particular choice of number of microstate classes to be fitted to the data. Therefore, our current pragmatic recommendation is that researchers should undertake the (tedious) effort to understand what changes in the choice of the number of classes imply for the obtained results, and select, present and discuss their results in a way that takes this uncertainty reasonably into account (e.g., Diezig et al. 2022). Future versions of the toolbox may provide users with updated means to address the problem.

Finally, we briefly want to connect the present paper to a series of other papers in this special issue. The paper by Nagabhushan Kalburgi et al. (eventually in this issue) systematically compared different automatic EEG preprocessing pipelines. EEG preprocessing standards were perceived as an important issue for microstate analysis at the microstate conference in 2022 in Bern, which also motivated this special issue. As a consequence of the conclusions of this paper, we have augmented the toolbox with artifact identification tools on the level of individual cluster maps. Another link exists to the paper by Koenig et al. (2023) that integrates EEG microstate template maps and their associated empirical findings across studies. The toolbox offers the possibility to use any of the template maps presented in these studies, and also the meta-microstate-template maps the author has obtained. On the other side, users of the toolbox can directly import the templates obtained in their analysis into the software presented in the mentioned paper, and objectively link their microstate templates to previously published templates and their associated findings. Another link exists to a paper by Kleinert et al. (2023a), where the authors tested for the retest-reliability and methodological consistency between microstate features obtained from different methods in a large sample of EEG recordings (n = 583). They found that backfitting with mean template maps yielded more reliable microstate features compared to backfitting with individual template maps, which led us to recommend this method. Furthermore, there are links to papers by Zanesco (in press) and Kleinert et al. (2023b), who report normative averages and intercorrelations of microstate parameters, respectively, providing a reference for future studies using the toolbox. We thus hope that this paper, in conjunction with the other papers in this special issue, fosters the collaborative spirit that characterized this conference.

In conclusion, we hope that our toolbox will help researchers from different fields to improve our knowledge on the temporal dynamics of the resting brain, and that the newly provided recommendations on critical decisions in the microstate analysis as well as the newly available functions will help to improve the current methodological standards of microstate research.

## Data Availability

The data used to demonstrate the toolbox usage is described in Sect. 4.2 and openly available here: https://osf.io/yqt7k/.
